# Antigenic cross-reactivity between *Schistosoma mansoni* and allergenic invertebrates putatively due to shared glycanic epitopes

**DOI:** 10.1038/s41598-020-59892-6

**Published:** 2020-02-25

**Authors:** Marwa H. El-Faham, Fatou Gai, Joseph E. Igetei, Sarah Richter, Franco H. Falcone, Gabi Schramm, Michael J. Doenhoff

**Affiliations:** 1 School of Life Sciences, University Park, University of Nottingham, Nottinghamshire, NG7 2RD UK; 2 Department of Medical Parasitology, Faculty of Medicine, Alexandria University, Alexandria, Egypt; 3 School of Pharmacy, Division of Molecular Therapeutics and Formulation, University of Nottingham, Nottingham, NG7 2RD UK; 4 Ministry of Health & Social Welfare, National Public Health Laboratories, Banjul, The Gambia; 5 Department of Animal and Environmental Biology, Faculty of Life Sciences, University of Benin, Benin City, Edo State Nigeria; 6 Parasitology Institute (Zoology), University of Hohenheim, Stuttgart, Germany; 7 Justus-Liebig-University Giessen, Institute for Parasitology, Biomedical Research Centre, Seltersberg (BFS) Schubertstr 81, 35392 Giessen, Germany; 8 Experimental Pneumology, Research Center Borstel, Airway Research Center North, Member of the German Center for Lung Research (DZL), Parkallee 22, D-23845 Borstel, Germany

**Keywords:** Biological sciences, Mass spectrometry

## Abstract

Previous studies have shown that rabbit IgG antibodies against *Schistosoma mansoni* egg antigens (SmSEA) cross-react with allergens in natural rubber latex, peanuts and grass and tree pollens. Here we describe antigenic molecules that cross-react with rabbit anti-*S*. *mansoni* IgG antibodies in extracts of the house dust mite (HDM) *Dermatophagoides farinae*, the Australian cockroach (ACR) *Periplaneta australasiae* and in the venom of the honey bee *Apis mellifera* (HBV). Tandem mass spectrometry identified the cross-reactive allergens as Der f 15 in HDM, two homologues of the *Periplaneta americana* cockroach allergen Cr-PI/Per a 3 in ACR and two isoforms of the allergen Api m 1 (phospholipase A2: PLA2) in HBV. Cross-reactive rabbit anti-SmSEA IgG antibodies eluted from the three invertebrate allergens reacted with *S*. *mansoni* egg antigens and variably with schistosome cercarial and worm antigens. Treatment of the electroblotted allergens with sodium metaperiodate abrogated most of the cross-reactivity of the rabbit anti-SmSEA antibodies, suggesting it was due to cross-reactive carbohydrate determinants (CCDs). Furthermore, analyses of the allergens’ amino acid sequences indicated that they had potential for both N- and O-linked glycosylation. A potential role for the CCDs shared by the schistosome and invertebrates in inducing an allergy-protective effect, as proposed by the hygiene hypothesis, is discussed.

## Introduction

In recent decades there has been a steep rise in the occurrence of allergic diseases, particularly in countries with well-developed health systems^1,2^. The ‘hygiene hypothesis’ is often offered as an explanation, i.e., due to an increase in healthy living conditions and/or prevention of infection by vaccinations and drugs, populations have suffered less from parasitic and microbial infections^3^ and development of human immune systems is now different from those of pre-hygienic, infection-rife eras^4^. A consequence can be an untimely, pathological immune response to environmental and air-borne antigens responsible for asthma and allergies, and which ‘normal’ human immune responsiveness would have perceived as innocuous.

In helminth-endemic countries in particular an inverse correlation has been observed between infection with helminths, including schistosomes, and allergic sensitization^5–7^ for which situation the hygiene hypothesis has been evoked as an explanation^8^.

Immune responses that cause allergic reactions are so-called Th2-type responses^9–11^ and Th2 cell-driven immunity is also induced by helminth infections^12–14^. Th2-biased immune responsiveness is however downregulated during chronic helminth infection in human hosts and this in turn has a suppressive effect on allergic sensitization in such individuals^15–17^. How this occurs has not been fully elucidated, though one explanation invokes activation of regulatory T cells (Tregs) and production of IL-10 and TGF-β, anti-inflammatory cytokines which have a dampening effect on atopic diseases such as allergy and asthma^18–20^.

‘Blocking antibodies’ may provide an alternative explanation for the hygiene hypothesis. Helminth infections induce the production of large amounts of non-specific IgE^21^, which could saturate Fcε receptors on mast cells and basophils and ‘block’ the cross-linking of such receptors by allergen-induced specific IgE antibody. There is, however, evidence against this possibility^22–24^. Nevertheless, the results presented here are consistent with an alternative possible ‘blocking antibody’ explanation since many proteins of plants and invertebrates (including helminths) are glycoproteins, the glycan epitopes of which can induce IgE antibodies^25–27^, some of which are known to be antigenically cross-reactive: so called cross-reactive carbohydrate determinants (CCDs)^28^. Glycan epitopes can also induce production of IgG antibodies^29^. The possibility that anti-*S*. *mansoni* IgG antibodies that cross-react with allergens could therefore block IgE-mediated allergic reactions is discussed as an explanation for the hygiene hypothesis.

We have so far demonstrated antigenic cross-reactivity, most likely due to CCDs, between *S*. *mansoni* and identified allergens in several different plants^30–32^. Those studies have been extended here to explore cross-reactivity between *S*. *mansoni* and allergenic invertebrates: namely, the house dust mite^33^, the cockroach^34^ and honey bee venom^35^.

## Material and Methods

Except when stated otherwise, all chemicals and reagents were of analytical grade and purchased from Sigma-Aldrich (now Merck), Poole, Dorset, England.

### Ethical approval

Experiments using mice for production of *S*. *mansoni* antigens and rabbits for production of antisera were approved by the Animal Welfare and Ethical Review Board of the University of Nottingham in which these materials were produced and the work was carried out in accordance with the regulations set out in the UK Animals (Scientific Procedures) Act, 1986, (Project licence numbers PPL 40/3024 and 40/3595). Animals were euthanized using a lethal dose of pentobarbitone anaesthetic.

### Preparation of soluble extracts of SmSEA

A Puerto-Rican isolate of *S*. *mansoni* was maintained by continuous passaging through the snail *Biomphalaria glabrata* and random-bred, CD1 strain mice.

*S*. *mansoni* soluble egg antigens (SmSEA) were extracted from eggs from the livers and intestines of infected mice harbouring adult worms as previously described^36^. The soluble extract was lyophilised and stored in 1 mg aliquots at −80 °C until use. It was used for immunoblotting at a concentration of 5 mg/ml protein. Extracts from *S*. *mansoni* worms and cercariae were prepared as described previously^37^.

### Preparation of allergen extracts

Powder of the house dust mite *Dermatophagoides farinae* (HDM) was incorporated (50 mg/ml) in phosphate-buffered saline (PBS), pH 7.4. The suspension was agitated gently for 30 minutes and centrifuged at 10,000 × g for 10 minutes, all at room temperature in a microcentrifuge. The supernatant containing 4.4 mg/ml protein was collected and kept in 500 µl aliquots at −20 °C in the short term (2 weeks) and at −80 °C in the longer term and they were used within 4–8 weeks of preparation (i.e., solution or suspension in buffer), after which fresh extracts were prepared for any subsequent experiments. Adults of a local infestation of cockroaches, identified as *Periplaneta australasiae* (ACR), were macerated in a mortar and pestle, suspended in an equal volume of PBS, agitated gently for 30 minutes at room temperature, sonicated on ice with 3 × 10 second pulses at 5,000 Hz separated by 10 second breaks and the suspension, in a 50 ml centrifuge tube, was centrifuged for 10 minutes at 4000 × g at room temperature utilising a swing-bucket centrifuge rotor. The supernatant containing 20 mg/ml protein was stored similarly to that of HDM extract. For production of bee venom (HBV), worker honey bees were anaesthetised with carbon dioxide gas and decapitated. The sting and venom sac of ~100 bees were gently pulled from the abdomen and placed in 1 ml ice-cooled distilled water, macerated with a plastic pipette tip, dispensed in 500 μl aliquots and centrifuged at 10,000 × g for 10 minutes. The supernatant contained 2.5 mg/ml protein and was stored as for other extracts.

Aqueous extracts of rubber latex (*Hevea brasiliensis*) (10.5 mg/ml), peanut (*Arachis hypogaea*) (33.4 mg/ml), tomato (*Lycopersicon esculatum*) (18.8 mg/ml), avocado (*Persea americana*) (22 mg/ml) and kiwi fruit (*Actinidia deliciosa*) (9.4 mg/ml) were prepared as described previously^30^. Estimates of protein concentrations in SmSEA and other extracts were done using the adapted^38^ Bio-Rad DC protein assay method (Bio-Rad Laboratories, Watford, UK), with bovine serum albumin (BSA) as standard.

### Preparation of rabbit antisera

Rabbits were laboratory-maintained under strict monitoring for health and hygiene and during the course of these studies there was no record of any occurrences of HDM, cockroach infestations or bees in the laboratories. Polyspecific antisera were raised against SmSEA by weekly immunization of New Zealand white rabbits as described previously^39^. Thus, a soluble extract of SmSEA prepared as described above containing approximately 5 mg/ml SEA was emulsified in an equal volume of complete Freund’s adjuvant and 1 ml of the emulsified homogenate was administered to rabbits in 0.1 ml quantities both intramuscularly (in both hind legs) and subcutaneously at multiple dorsal sites. The rabbits were serially bled via an ear vein weekly and injections were continued until a strong antibody response was obtained against the SEA immunogen in immunoelectrophoresis. Rabbits were terminally anaesthetised and exsanguinated by cardiac puncture and sera were collected and stored at −20 °C. Sera from 2 immunized rabbits, BR84 and 1025Z, were individually used here for experiments on the house dust mite and on the cockroach and honey bee venom, respectively.

Honey bee venom phospholipase A2 was purchased from Sigma-Aldrich (now Merck), UK, and a polyclonal rabbit antiserum raised by injecting 50 µg that had been dissolved in 1 ml PBS and emulsified with 1 ml Freund’s adjuvant in several subcutaneous sites. The injections were repeated weekly for 5 weeks, after which the rabbit was exsanguinated and the serum aliquoted and stored at −20 °C until required.

Sera from rabbits injected with Freund’s adjuvant alone, developed as scheduled above, were used as control normal rabbit sera (NRS).

### One-dimensional sodium dodecyl polyacrylamide gel electrophoresis (SDS-PAGE) and western immunoblotting

SDS-PAGE methodology was adapted^40^ and performed as described^41^ using 12% or 8% polyacrylamide gels. SDS-PAGE gels were run using a Bio-Rad Mini Protean II electrophoresis system (Bio-Rad Laboratories, California, USA). Western immunoblotting was adapted and performed as described previously^30,42^. SDS-PAGE-resolved proteins were transferred to nitrocellulose membranes (NCM) and probed with rabbit antisera diluted 1:100 in Tris-buffered saline with 0.5% v/v Tween 20 (TBST) overnight at 4 °C as the primary antibody, followed by 2 hours at room temperature in a solution of horse radish peroxidise (HRP)-conjugated goat anti-rabbit IgG antibodies (Sigma Aldrich, now Merck, UK) diluted 1:1000 in TBST as the secondary antibody. The immunoblots were developed using 4-chloro-1-naphthol substrate (Sigma Aldrich, now Merck, UK) as described by the manufacturer. At least three replicates of SDS-PAGE gels and immunoblots have been done for cross-reactive experiments in the present work.

### Staining and purification of electrophoresed proteins in SDS-PAGE

SDS-PAGE gels containing electrophoresed proteins were stained with Coomassie blue for protein visualization using SimplyBlue SafeStain (Invitrogen, Carlsbad, CA) according to the manufacturer’s instructions. Partial purification of proteins was achieved by excision of the bands of interest (identified by matching with western blots displaying the immunoreactivities of interest) from a stained gel, followed by overnight elution in 10% SDS, 0.06 M Tris-HCl, pH 7.0, buffer at 37 °C^30,31^. Solutions containing eluted proteins were concentrated using Amicon ultra centrifugal filters, 3000 molecular weight cut-off (Millipore, Corrigtwohill, Co. Cork, Ireland) and re-electrophoresed in a second SDS-PAGE as recently described^31,43^. Purified, concentrated proteins were stored at −20 °C.

### Purification of cross-reactive anti-SmSEA IgG antibodies by acid-elution

Anti-SmSEA antibodies that were cross-reactive with molecules from the allergen extracts were purified by acid-elution using an adapted method^44^ modified as described^30^ (Supplementary Fig. S1). Protein extracts were individually loaded into a single, wide lane in a SDS-PAGE gel and electro-transferred to a nitrocellulose membrane. The membrane was blocked and incubated with the primary rabbit antiserum as described above. 1 cm parallel longitudinal nitrocellulose paper strips were cut from each edge of the immunoblot, washed and incubated with horse-radish peroxidase-conjugated goat anti-rabbit IgG antibodies and chromogenically stained. The position of the target immune-complex in the undeveloped main part of the immunoblot was determined by realigning the two stained strips against the sides of the membrane. A horizontal strip containing the immune-complex of interest was then cut from the blot and the antibodies were eluted therefrom in 1 ml of 0.1 M glycine buffer, pH 2.8, gently agitated at room temperature for 10 minutes. The eluting buffer was collected and neutralised using 1 M Tris, pH 8.0, and stored at −20 °C. The strip was washed three times in PBS, each for 5 minutes with gentle agitation. The process of incubating the strip with primary antibody, washing and antibody-elution using low pH buffer was repeated up to 4 times.

### Treatment of proteins electroblotted onto NCM with sodium metaperiodate

The technique was adapted^30^ and performed as described earlier^45,46^. Briefly, nitrocellulose membranes carrying electroblotted antigens were incubated for 1 hour in 10 mM sodium metaperiodate dissolved in 0.05 M sodium acetate buffer, pH 4.5, in the dark at room temperature. Controls were treated in 0.05 M sodium acetate buffer, pH 4.5 under similar conditions, but without the metaperiodate. The membranes were washed three times in TBST and the process for western immunoblotting was then continued with blocking, incubation with primary and secondary antibodies and development methods as described above.

### Tandem mass spectrometry (TMS) analysis of protein samples and data analysis

Protein bands excised from Coomassie blue-stained gels were subjected to nanoflow Liquid Chromatography Electrospray Ionization TMS (nLC-ESI MS/MS)^47^ as described elsewhere^48^. The analysis was done by the BSRC Mass Spectrometry and Proteomics Facility, University of St Andrews, UK. The MS/MS data were analysed using the Mascot algorithm (Matrix Science), against the NCBInr database (August 2016). A protein was accepted as identified if it had two or more peptides with Mascot Ion Scores above the Identity Threshold (*P* < 0.05). TMS-identified proteins were further analysed using the BLAST Tool (http://blast.ncbi.nlm.nih.gov/Blast.cgi) and the pairwise sequence alignment tool (http://www.ebi.ac.uk/Tools/psa/). The prediction of potential glycosylation sites on TMS-identified allergens was done using the CBS Software Prediction Servers NetNGlyc and NetOGlyc (http://www.cbs.dtu.dk/services/NetNGlyc/ & http://www.cbs.dtu.dk/services/NetOGlyc/).

## Results

### Resolution of HDM, ACR and HBV invertebrate extracts by SDS-PAGE and reactivity of rabbit anti-SmSEA IgG antibodies thereon

**Figure 1:**
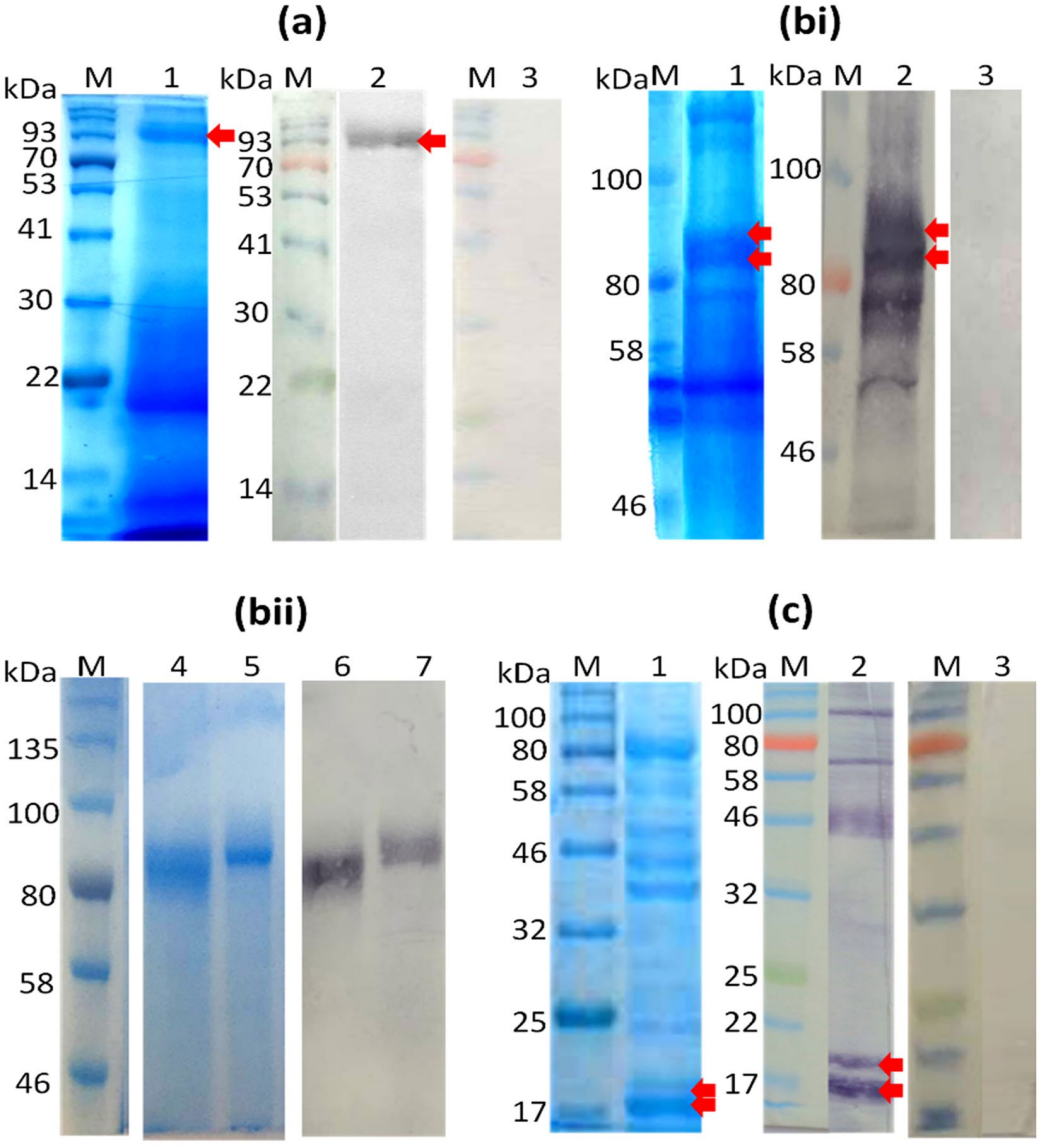
Coomassie blue-stained SDS-PAGE and western immunoblots of crude invertebrate extracts, and purified proteins from house dust mite (*D*. *farinae)*, Australian cockroach (*P*. *australasiae*) and honey bee (*A*. *mellifera*) venom, probed with anti-*S*. *mansoni* SEA antisera. (**a**) *D*. *farinae* extract (44 µg/lane): lane 1, Coomassie blue-stained gel; lanes 2,3, Western immunoblots; (**bi**) *P*. *australasiae* extract (200 µg/lane): lane 1, Coomassie blue-stained gel; lanes 2,3 immunoblots; (**bii**), *P*. *australasiae* purified proteins (10.6 µg/lane): Coomassie blue-stained purified *P*. *australasiae* protein at ~85 kDa (lane 4) and ~88 kDa (lane 5); immunoblot of purified cockroach proteins of ~85 kDa (lane 6) and ~88 kDa (lane 7). (**c**) Honey bee venom (25 µg/lane): lane 1, Coomassie blue-stained gel; lanes 2,3, Western immunoblots. 12% SDS PAGE gels were used in panels (**a,c**) and 8% SDS PAGE gels in panels (bi) and (bii). Blots were probed with anti-*S*. *mansoni* SEA antiserum BR84 on lane 2 in (**a**); anti-SmSEA antiserum 1025Z on lane 2 in (**bi**), on lanes 6 and 7 in (**bii**) and on lane 2 in (**c**), or normal rabbit serum on lanes 3 in (**a**), (**bi**) and (**c**). Cross-reactive protein bands at >90 kDa (**a**), ~85 kDa & ~88 kDa (**bi** and **bii**) and ~17 kDa & 19 kDa (**c**) that were selected for TMS are arrowed. M = protein molecular size marker. Supplementary Fig. S5 shows uncropped blot images for lane 2 (**bi**) and lanes 4–7 (**bii**).

Figure 1 shows a composite of results of electrophoretic resolution of material from 3 allergenic invertebrates by one-dimensional SDS-PAGE, followed by staining with Coomassie blue or electroblotting on NCM and probing with a rabbit anti-SmSEA antiserum. Figure 1a comprises the results on the HDM extract electrophoresed in a 12% polyacrylamide gel. Lane 1 in the Coomassie blue-stained gel shows a protein band of molecular size >90 kDa which appears to be the sole reactant with IgG antibodies in rabbit BR84 anti-SmSEA antiserum in the immunoblot (Fig. 1a, lane 2). Normal rabbit serum gave no reactivity against HDM (Fig. 1a, lane 3). A sample of the >90 kDa band in the Coomassie-stained gel (indicated by a red arrow against lane 1, Fig. 1a) was submitted for TMS analysis.

Fig. 1bi, lane 1 shows the ACR extract in a Coomassie blue-stained 8% polyacrylamide gel. Lane 2 in Fig. 1bi is an immunoblot of the electrophoresed ACR probed with anti-SmSEA 1025Z IgG antibodies. At least four molecules between 46 kDa and 100 kDa were cross-reactive with the anti-SmSEA antibodies (Fig. 1bi, lane 2). No reactivity against ACR was observed with normal rabbit serum (Fig. 1bi, lane 3). Gel slices containing two molecules estimated to be 85 kDa to 88 kDa were excised from a replicate of the gel in lane 1 and the proteins were eluted from the respective gel slices by overnight incubation in buffer. The eluted proteins were re-electrophoresed for Coomassie blue-staining (Fig. 1bii, lanes 4 and 5). After immunoblotting the purified molecules each reacted with IgG antibodies in the rabbit anti-SmSEA 1025Z antiserum (Fig. 1bii, lanes 6 and 7) and samples of each excised from the acrylamide gels were therefore subjected to TMS analysis.

In HBV several proteins with molecular sizes ranging from <20 kDa to >100 kDa reacted with the rabbit anti-SmSEA 1025Z serum (Fig. 1c). Two of these molecules of ~17 kDa and ~19 kDa were purified and found to react with a rabbit antiserum raised against HBV PLA2 (Supplementary Fig. S2). These two molecules were taken forward for analysis by TMS.

### Characterization of schistosome cross-reactive invertebrate molecules by tandem mass spectrometry (TMS)

Molecules in the invertebrate extracts that cross-reacted with anti-SmSEA antibodies were selected for further analysis by mass spectrometry, the selected proteins having been excised from well-washed Coomassie blue-stained gels. The results of TMS on the sole cross-reactive >90 kDa protein in the HDM extract (Fig. 1a) are summarized in Supplementary Table S1 and they indicate that a known HDM allergen, Der f 15 (gi: 5815436), was present. The TMS did not detect any peptides other than those from Der f 15.

The results of TMS analysis of two purified ACR proteins of estimated size ~85 kDa and ~88 kDa (Fig. 1bii, lanes 4 and 5) are given in Supplementary Tables S2 and S3 and they indicate both protein bands contained peptides present in the sequence of protein Cr-PI, allergen Per a 3 (gi: 284518363) from *Periplaneta americana*.

TMS analysis of the ~17 kDa and ~19 kDa HBV molecules (Fig. 1c) confirmed that both protein bands contained HBV phospholipase A2 (gi: 5627, GenBank: CAA34681.1), also known as the allergen Api m 1 (Supplementary Tables S4 and S5).

### Reactivity and periodate-sensitivity of acid-eluted antibodies

**Figure 2:**
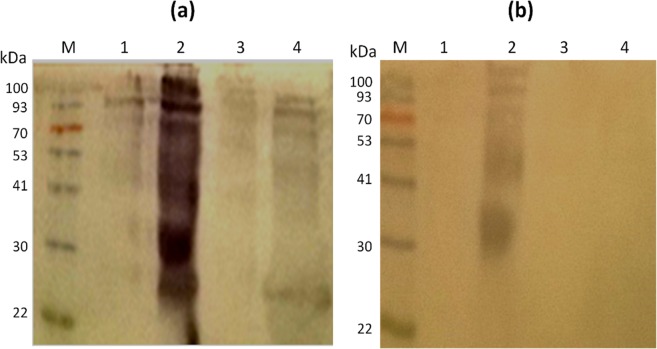
Western blot of *D*. *farinae* and *S*. *mansoni* extracts probed with rabbit BR84 IgG antibodies eluted from a >90 kDa *D*. *farinae* molecule. (**a**) Eluted antibodies were used to probe: 1, *D*. *farinae* extract (44 µg/lane); 2, SmSEA (20 µg/lane); 3, *S*. *mansoni* cercarial homogenate (20 µg/lane); 4, *S*. *mansoni* worm homogenate (20 µg/lane). (**b**) The same as (**a**), but treated with 10 mM Na-metaperiodate for one hour prior to application of primary antibody. M = protein molecular size marker.

Supplementary Fig. S1 illustrates how rabbit anti-SmSEA BR84 antibodies that were cross-reactive with the >90 kDa HDM molecule were purified by acid-elution from electroblotted HDM extract. This method was also used to purify anti-SmSEA IgG antibodies that cross-reacted with cockroach and bee venom antigens. Eluted antibodies were obtained at concentrations that ranged from 1 to 1.3 mg/ml. The acid-eluted antibodies were used to probe electroblots of extracts from HDM and from different stages of the *S*. *mansoni* life-cycle and the results are shown in Fig. 2a.

Reactivity of the eluted antibodies against HDM was relatively weak, including against the >90 kDa molecule itself (Fig. 2a, lane 1). There was however intense reactivity against the constituents of SmSEA over a wide range of molecular size (Fig. 2a, lane 2), though only relatively low reactivity against *S*. *mansoni* cercarial or adult worm antigens (Fig. 2a, lanes 3 and 4). No reactivity was observed on any of the parasite extracts with a normal rabbit serum (Supplementary Fig. S3).

Figure 2b shows the result of treatment of a replicate NCM film carrying the same electroblotted antigens as in Fig. 2a, with 10 mM Na-metaperiodate for 1 hour before incubating the film with the anti-HDM eluted antibodies. There is no antibody reactivity against the HDM extract or the *S*. *mansoni* cercarial and worm extracts, and the intensity of reactivity against the SmSEA is much reduced. Supplementary Fig. S4 illustrates SDS-PAGE gels of resolved proteins in the three stages of *S*. *mansoni* life-cycle extracts used in blots in Fig. 2.

**Figure 3:**
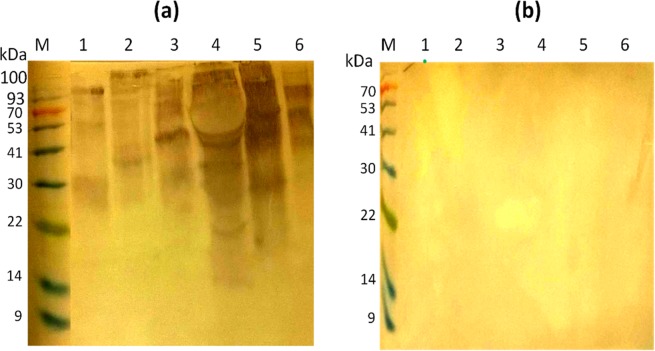
Western blots of extracts of allergenic plants probed with rabbit BR84 anti-SmSEA antibodies eluted from a >90 kDa *D*. *farinae* molecule. (**a**) Eluted antibodies were used to probe: 1, *D*. *farinae* (44 µg/lane); 2, latex (157.5 µg/lane); 3, tomato (188 µg/lane); 4, peanut (167 µg/lane); 5, avocado (220 µg/lane); 6, kiwi fruit (94 µg/lane). (**b**) The same as (**a**), but after treatment with 10 mM Na-metaperiodate for one hour prior to application of primary antibody. M = protein molecular size marker.

Results in Fig. 3a show the reactivity of BR84 antibodies that had been acid-eluted from the >90 kDa HDM against the HDM extract and against extracts from a variety of plants. The reactivity against HDM was relatively weak as in Fig. 2a, lane 1, but there was reactivity against a range of antigens in the different plant extracts, with relatively intense reactivity against peanut and avocado. Treatment of the electroblotted extracts with 10 mM Na-metaperiodate prior to incubation with the acid-eluted rabbit antibodies resulted in complete abrogation of the antibody cross-reactivity against all the extracts (Fig. 3b). No reactivity was observed on any of the plants with a normal rabbit serum (Supplementary Fig. S3).

**Figure 4:**
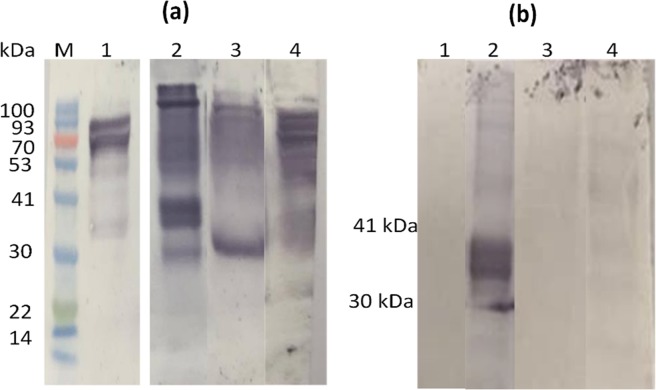
Western immunoblots of *P*. *australasiae* and *S*. *mansoni* extracts probed with rabbit 1025Z anti-SmSEA IgG antibodies eluted from ~85 kDa and ~88 kDa *P*. *australasiae* molecules. (**a**) Eluted antibodies were used to probe: 1, *P*. *australasiae* extract (200 µg/lane); 2, *S*. *mansoni* SEA (20 µg/lane); 3, *S*. *mansoni* worm homogenate (20 µg/lane); 4, *S*. *mansoni* cercariae homogenate (20 µg/lane). (**b)** The same as (**a**) but after treatment with 10 mM Na-metaperiodate for one hour. M = protein molecular size marker. Supplementary Figs. S6 and S7 show uncropped blot images for lanes 1–4 of panels (a,b).

Figure 4a shows the reactivity of 1025Z antibodies eluted from both the ~85 kDa and the ~88 kDa ACR molecules against a variety of antigens in extracts from different life cycle stages of *S*. *mansoni*. (Elution from both molecules together was justified since the TMS results indicated that both were orthologues of the cockroach allergen Per a 3). The eluted anti-85/88 kDa ACR antibodies reacted relatively intensely against those two molecules in the ACR extract (Fig. 4a, lane 1). Likewise, reactivity of anti-85/88 kDa ACR antibodies against SmSEA was also relatively intense (Fig. 4a, lane 2), as well as against *S*. *mansoni* worm and cercarial molecules (Fig. 4a, lanes 3 and 4 respectively), all of which showed more intense staining than that of the eluted anti-HDM >90 kDa antibodies in Figs. 2, 3. After Na-metaperiodate treatment neither the 85 kDa nor 88 kDa ACR molecules reacted with the acid-eluted anti-ACR 85/88 kDa antibodies (Fig. 4b, lane 1). Cross-reactivity of these acid-eluted antibodies against adult schistosome worm and cercarial antibodies was also almost completely abrogated (Fig. 4b, lanes 3 and 4). In periodate-treated SmSEA only a broad band between the 32 and 46 kDa markers remained reactive with these antibodies (Fig. 4b, lane 2).

**Figure 5:**
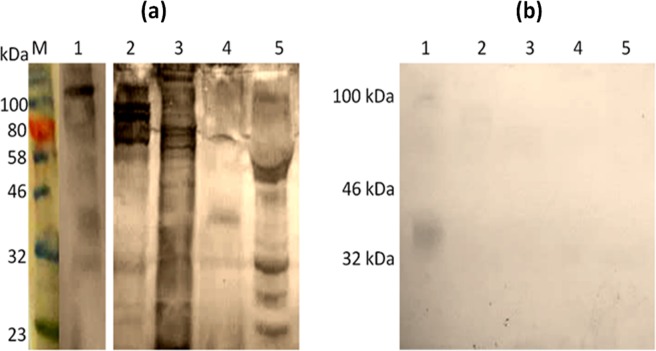
Western immunoblots of extracts of *S*. *mansoni* and allergenic plants probed with rabbit 1025Z IgG antibodies eluted from ~17–19 kDa bee venom molecules. (**a**) Eluted antibodies were used to probe: 1, *S*. *mansoni* SEA (20 µg/lane); 2, *S*. *mansoni* worm homogenate (20 µg/lane); 3, *S*. *mansoni* cercariae homogenate (20 µg/lane); 4, latex (157.5 µg/lane); 5, peanut (167 µg/lane). (**b**) The same as (**a**) but after treatment with 10 mM Na- metaperiodate for an hour before incubation with primary antibody. M = protein molecular size marker. Supplementary Fig. S8 shows uncropped blot images for lanes 1–5 (**a**).

Figure 5 shows the results of probing different extracts with 1025Z antibodies eluted from the ~17 kDa and ~19 kDa HBV molecules. As with the antibodies eluted from the two ACR molecules, the two HBV molecules were not treated separately here because they were shown by TMS to be isoforms of PLA2/Api m 1. The eluted antibodies reacted against a variety of molecules in SmSEA and *S*. *mansoni* worm and cercarial antigens (Fig. 5a, lanes 1, 2 and 3 respectively) and against extracts of latex and peanut (Fig. 5a, lanes 4 and 5 respectively). In SmSEA the most intense reactivity was against a ~100 kDa molecule. Treatment of the NCM with 10 mM Na-metaperiodate prior to application of the acid-eluted primary antibodies resulted in abrogation of all the cross-reactivity observed in Fig. 5a, except for a residual faint smudge of reactivity between 32 kDa and 46 kDa in SmSEA.

### Identification of potential glycosylation sites in amino-acid sequences of invertebrate allergens

Bioinformatic analysis of the potential N-linked and O-linked glycosylation sites for the 3 invertebrate allergenic molecules are indicated in Supplementary Tables S6 to S10. HDM Der f 15 has three N-linked sites (Supplementary Table S6) and 54 O-linked sites (Supplementary Table S7). ACR Per a 3 has two N-linked sites (Supplementary Table S8) and no O-linked sites. HBV Api m 1 has one N-linked site (Supplementary Table S9) and three O-linked sites (Supplementary Table S10).

## Discussion

Previous studies investigating antigenic cross-reactivity between *S*. *mansoni* and allergens from different organisms have demonstrated cross-reactivity between the helminth and the allergens Hev b 7 from natural rubber latex *Hevea brasiliensis*^30^, Ara h 1 from the peanut *Arachis hypogaea*^31^ and a total of five distinct allergenic molecules in plant pollens^32^. The aforementioned studies have now been extended to exploration of cross-reactive antigenic relationships between *S*. *mansoni* and extracts from three invertebrates which are important causes of allergic reactions, particularly asthma and hypersensitive skin reactions.

Hypersensitive pathological immune responses against the constituents of such invertebrates constitute serious world-wide health problems. Thus, as stated by Calderon *et al*.^33^, ‘The house dust mite (HDM) is a major perennial allergen source and a significant cause of allergic rhinitis and allergic asthma. Prevalence data for HDM allergen sensitization vary from 65 to 130 million persons in the general population worldwide to as many as 50% among asthmatic patients’. Likewise cockroaches, established as an important cause of allergies over 50 years ago^49^, represent one of the most common sources of indoor allergens worldwide, with 40–60% of patients with asthma in urban and inner-city areas possessing IgE antibodies to cockroach allergens^34,50^. Estimates of the prevalence of systemic (anaphylactic) reactions to hymenoptera stings in adult Americans range from 0.5% to 3.3%^51^ while European epidemiological studies report a prevalence of systemic reactions between 0.3% and 7.5%^52^.

Der f 15, a >90 kDa HDM molecule found here to be the sole reactant with anti-SmSEA antibodies, is a chitinase. Together with the chitinase-like Der f 18, Der f 15 is considered a minor allergen for humans^53,54^, but a major allergen for dogs^55^. Most dogs with HDM-associated atopic dermatitis had IgE antibodies to two proteins of apparent molecular weight 98 kDa and 109 kDa^55^. The two proteins had identical amino acid sequences which predicted a protein of 63.2 kDa^55^ and which TMS analysis for the >90 kDa HDM protein studied here gave as 63.8 kDa (Supplementary Table S1). Rabbit anti-SmSEA IgG antibodies here reacted against only one molecule between 93 kDa and 100 kDa, presumably the smaller of the two molecules identified as Der f 15 by McCall *et al*.^55^,. The 30–40 kDa difference between the size predicted by the 555 amino acid-long sequence and that indicated in their immunoblots was attributed to extensive glycosylation^55^. Our analysis of potential sites for glycosylation indicates that Der f 15 may be glycosylated via numerous O-linked sites, particularly in a threonine/proline-rich section of its sequence (amino acids 415–498) found towards the C-terminal end of the molecule and three N-linked sites, though only one N-linked site was predicted in the analysis by McCall *et al*.^55^.

Alignment of linear amino-acid sequences of Der f 15 with those of three SmSEA molecules which have also previously been implicated in antigenic cross-reactivity with other allergen molecules^30–32^ shows only 10–15% identity (data available on request). The reactivity of the eluted antibodies against the HDM extract and schistosome egg antigens was abrogated by periodate-treatment, except for some reactivity that remained in the SmSEA. Thus, the cross-reactivity of anti-SmSEA antibodies with Der f 15 seems likely to be due to recognition of glycan rather than peptide epitopes. Amid a wide-ranging smudge of cross-reactivity, three molecules of ~100 kDa, 30–35 kDa and 25–28 kDa in SEA reacted with antibodies eluted from Der f 15. These SEA molecules may be, respectively, the immunodominant *S*. *mansoni* egg antigens kappa-5^56^, IPSE/alpha-1^57^ and omega-1^58^. All three SmSEA molecules are N-glycosylated and their respective patterns of glycosylation have been elucidated: kappa-5^59^, IPSE/alpha-1^60^ and omega-1^61^. O-linked glycans have not been found on the above-mentioned SEA proteins, so some of the cross-reactivity of the rabbit anti-SmSEA antibodies with Der f 15 may be associated with glycosylation of the N-linked site(s) on the latter molecule. None of the other SmSEA molecules reacting with the acid-eluted anti-Der f 15 antibodies have been investigated, but O-linked glycans thereon^62^ may have induced antibodies cross-reactive with Der f 15. The notion that the cross-reactivity between Der f 15 and antigens in SmSEA is due to shared cross-reactive carbohydrate determinants (CCDs)^25,28^ is supported by the observation here that the anti-SmSEA antibodies eluted from Der f 15 reacted against not only SmSEA, but also against various plant extracts. Furthermore, all the reactivity against the plant extracts was abrogated completely by prior treatment of the material electroblotted onto the nitrocellulose film with Na-metaperiodate, which destroys carbohydrate determinants^63,64^.

For this work a local infestation of the Australian cockroach *P*. *australasiae* provided a convenient, cost-free source of cockroach-derived material. At least four molecules in the cockroach extract, mainly between 46 kDa and 100 kDa, were found to be cross-reactive with rabbit anti-SmSEA antibodies in the present work. TMS analysis of two of the SmSEA cross-reactive molecules of ~85 kDa and ~88 kDa indicated they both contained peptides with sequences identical to those found in *P*. *americana* protein Cr-PI, allergen Per a 3 (gi: 284518363)^65,66^. *Periplaneta americana* and *Blatella germanica* are deemed responsible for most allergic reactions to cockroach with twelve *P*. *americana* allergens known^34^ (http://www.allergen.org/). Unsurprisingly, at the time of writing a Cr-PI/Per a 3 homologue of *P*. *australasiae* is not amongst the 75 sequences from this cockroach species currently in the NCBI protein database, compared with a listing of >2250 *P*. *americana* proteins, including ~10 sequences for Cr-PI and/or Per a 3. Further discussion of the *P*. *australasiae* molecule studied here is therefore necessarily restricted to what is known about its *P*. *americana* orthologue.

Cr-PI/Per a 3 is an important human allergen as partially purified fractions of Cr-PI elicited skin reactions in 73% of patients who were sensitive to the crude *P*. *americana* extracts^67^. This qualifies Per a 3 as a major allergen^68^. Four isomers of Per a 3 are known with molecular sizes ranging from 46–79 kDa based on amino acid sequence^65,66,69^. The sizes of native forms of the allergen molecules in preparations of Cr-PI were estimated to be 72 kDa and 78 kDa^67^ while the molecular weights of mature Per a 3 calculated from amino acid sequences of two recombinant clones were given as 75.5 kDa and 79.3 kDa^66^. The former two values for the native *P*. *americana* molecules are lower than the values of 85 kDa and 88 kDa estimated for native forms of the two schistosome cross-reactive proteins in the *P*. *australasiae* extract studied here, which may be due to differences between the two cockroach species. The two isoforms of Per a 3 studied by Wu *et al*.^66^, were both found to have two sites for potential N-linked glycosylation, as was found by the analysis here. As with Der f 15 above, a low degree of amino acid sequence similarity found by pair-wise comparisons between Per a 3 and three immunodominant antigens of SmSEA: kappa-5, IPSE/alpha-1 and omega-1 (data available on request) suggests the antigenic cross-reactivity is unlikely to be due to shared peptide sequences. The identity of the *S*. *mansoni* worm and cercarial antigens that cross-reacted with the *P*. *australasiae* Per a 3 homologue is unknown, but all that cross-reactivity, apart from a broad band in SmSEA between 32 kDa and 46 kDa, disappeared as a result of periodate-treatment.

Comparison of the results from Der f 15 and Per a 3 indicate that the anti-SmSEA antibodies eluted from Der f 15 reacted strongly against SmSEA, but much less intensively against parasite cercarial and worm antigens and Der f 15 itself. In contrast, antibodies eluted from Per a 3 reacted well against molecules in all 3 schistosome extracts as well as the Per a 3 double band, this despite the fact that Der f 15 is potentially more heavily glycosylated than Per a 3. There is a large difference between the calculated amino-acid mass of Der f 15 (63.882 kDa) and its mass when resolved by electrophoresis (98 kDa), but this difference was much lower for Per a 3 which has a sequence-calculated mass of 82.3 kDa and of 85–88 kDa when derived by electrophoresis. In this study, analysis of the Der f 15 amino-acid sequence indicated the molecule has many more potential sites for O-linked glycosylation than Per a 3, which is predicted to have none, but otherwise little is known about these two molecules. The different reactivities of the anti-SmSEA antibodies eluted from Der f 15 with extracts of the three schistosome stages may be consistent with the previous findings of the presence of multifucosylated antennae in mature eggs and miracidia and its absence in worms^62^, indicating that this cross-reactivity may be due to α3-core fucose residues.

Twelve honey bee (*Apis mellifera*) allergens are currently listed (http://www.allergen.org/). Phospholipase A2 (PLA2) was identified as a major allergen early on^70^ and it constitutes 12% of the material in honey bee venom^71^. Three isoforms of PLA2 have been identified, of estimated molecular sizes 16 kDa, 18 kDa and 20 kDa: the larger two molecules are glycosylated while the 16 kDa is not^72^. The two isoforms of estimated size 17 kDa and 19 kDa observed here that are antigenically cross-reactive with anti-SmSEA antibodies may thus correspond with the two larger isoforms described by Altmann *et al*.^72^, N-glycosylated via just one asparagine residue situated at position 13^73^ and Asn 41 as described here (Supplementary Table S9). In the present study, anti-SmSEA antibodies eluted from electroblots of the 17/19 kDa isoforms of PLA2 reacted against a variety of schistosome antigens, but again nearly all this cross-reactivity did not survive periodate treatment. One ~100 kDa molecule in SmSEA was particularly reactive with the eluted anti-17/19 kDa antibodies, and this may be kappa-5^56^. As with Der f 15 and Per a 3, pairwise comparison of the amino acid sequences indicates PLA2 and kappa-5 have little identity (~6%) or similarity (~12%) (data available on request), indicating that glycan epitopes are probably responsible for the antigenic cross-reactivity between the two molecules and anti-schistosome antibodies.

Glycan residues on both the larger-sized PLA2 variants were observed to contain fucose, mannose and N-acetylglucosamine, while the largest had, in addition, N-acetylgalactosamine^72,74^. Xylose and fucose are constituents of many plant and invertebrate glycoproteins^27,75^. Xylose is, however, generally considered absent in PLA2^74,76^, though paradoxically a rabbit antiserum specific for an oligosaccharide-linked xylose residue was found to react with PLA2^77^ and Altmann *et al*.^72^ found traces of this monosaccharide, which they considered an impurity, in their preparation of the 20 kDa isoform. The antigenic cross-reactivity between PLA2 and the putatively identified SmSEA antigen kappa-5 is therefore potentially interesting: kappa-5 has four potential N-glycosylation sites carrying triantennary glycans composed of a core region that is both difucosylated and xylosylated^59^, but the core α3-fucose residues may be primarily responsible for the cross-reactivity. The alpha 1,3 fucosylated glycan residue of PLA2/Api m 1 is cross-reactive with plant glycoproteins^75^, as also illustrated here by reactivity of anti-SmSEA antibodies eluted from PLA2 with a ~46 kDa band in a rubber latex extract and several proteins in peanut. The 46 kDa latex molecule could be Hev b 7, shown previously to be antigenically cross-reactive with *S*. *mansoni*^30^ and which might be responsible for reactivity to natural rubber latex in some patients with insect venom allergy^78^.

Antigenic reactivity of a band of 32–46 kDa in SmSEA survived periodate-treatment to some extent in the present study. The antigen responsible for this is likely IPSE/alpha-1^57^, a molecule which binds to immunoglobulins in a non-immunological manner^79^. Apparent periodate-resistance of the antigenicity of IPSE/alpha-1 has been discussed elsewhere^32^. The supposition that the cross-reactivity between rabbit anti-SmSEA antibodies and the allergens is due to shared CCDs is supported by results (personal communication: R Hokke, 13 July 2018) showing that antibodies in the rabbit sera were highly reactive on a microarray of synthetic N-linked glycans with α3-fucose containing core structures, alone or in combination with β2-xylose (see^80,81^ for principles of the method). Core α3-fucosylation is characteristic of the N-linked glycans of IPSE/alpha-1^60^ and omega-1^61^, while the core of kappa-5 N-linked glycans is modified by both α3-fucose and β2-xylose^59^. A recent study^81^ has confirmed that core β-1,2-xylose- and α-1,3-fucose-specific antibody responses are associated with *S*. *mansoni* infections in rural environments.

The present study has used rabbit anti-*S*. *mansoni* IgG antibodies to demonstrate antigenic cross-reactivity between antigens and allergens from three invertebrates, with potential involvement of cross-reactive carbohydrate determinants (CCDs). The existence of antigenic cross-reactivity between plants, invertebrates and molluscs, attributable to CCDs, has been known for a long time^77,82,83^. This is, however, seemingly the first time the carbohydrate determinants on a helminth’s antigens have been shown to be cross-reactive with those of known invertebrate allergens. In the context of the present results, dogs subjected to allergen-specific immunotherapy for atopic dermatitis produced IgG antibodies (isotype not specified) against a 98 kDa *D*. *farinae* molecule, presumed to be Der f 15^84^ and specific immunotherapy with whole bee venom resulted in an increase in the ratio of anti-PLA2 IgG4:IgE in serum^85^. High IgG levels, including anti-CCD-specific IgG4, are a frequent outcome of successful specific immunotherapy for allergies^86^ and pollen immunotherapy^29^. High levels of IgG4 are also found in subjects with chronic helminth infections^87,88^ and a recent study in Uganda on the relationship between immune responses to *S*. *mansoni* and allergy found that: ‘total and allergen-specific IgG4/IgE ratios were mostly inversely associated with atopy, implying that the regulatory role of IgG4 against allergy might best be assessed relative to IgE'^89^. Thus, it could be envisaged from our results that anti-schistosome anti-CCD IgGs induced by schistosome antigens that share identical epitopes with invertebrate allergens may induce IgGs that elicit a blocking effect on allergenic IgE.

IgG4 antibodies are postulated to inhibit immediate hypersensitivity reactions by two possible mechanisms: sequestering antigen and thus preventing cross-linking of receptor-bound IgE, and/or inhibiting IgE-facilitated allergen presentation to antigen presenting B cells^90^. An alternative hypothesis involves co-aggregation of FcεRI to FcγRIIb, e.g. by immune complexes, which has been shown to block basophil and mast cell activation *in vitro*^91–94^. The effect has been confirmed *in vivo* using chimeric human-cat Fcγ-Fel d 1 allergen fusion proteins^95^.

Future experiments may seek to investigate potential blocking effects of cross-reactive anti-*S*. *mansoni* IgG on different allergen-induced IgE antibodies and their potential interference with early- and/or late-phase allergic reactions. A perhaps surprising statistic is that of the molecules in plants and invertebrates that so far happen to have been studied because they are cross-reactive with anti-SmSEA antibodies, all ten are known allergens; namely Hev b 7 in natural rubber latex^30^, Ara h 1 in peanut^31^, Bet v 1, Bet v 6.01 and a glutathione S-transferase in birch tree pollen^32^, the GST having been subsequently shown to be an allergen^96^, Phl p 1 and Phl p 5 in Timothy grass pollen^32^, and Der f 15, Per a 3 and Api m 1 in this study. The rabbit anti-SmSEA antibodies used in this series of studies however cross-reacted with more than one molecule in the extract of peanut^31^ and more than five plant pollen molecules^32^. If many of these are also found to be known allergens, CCD-dependent cross-reactivity between *S*. *mansoni* and allergens might be quite commonplace.

## Supplementary information

supplementary materials.

## Data Availability

The data generated and/or analysed during the current study are available from the corresponding author on reasonable request.
