# Author Correction: Antigenic cross-reactivity between *Schistosoma mansoni* and allergenic invertebrates putatively due to shared glycanic epitopes

**DOI:** 10.1038/s41598-020-65548-2

**Published:** 2020-05-20

**Authors:** Marwa H. El-Faham, Fatou Gai, Joseph E. Igetei, Sarah Richter, Franco H. Falcone, Gabi Schramm, Michael J. Doenhoff

**Affiliations:** 1 School of Life Sciences, University Park, University of Nottingham, Nottinghamshire, NG7 2RD UK; 2 Department of Medical Parasitology, Faculty of Medicine, Alexandria University, Alexandria, Egypt; 3 School of Pharmacy, Division of Molecular Therapeutics and Formulation, University of Nottingham, Nottingham, NG7 2RD UK; 4 Ministry of Health & Social Welfare, National Public Health Laboratories, Banjul, The Gambia; 5 Department of Animal and Environmental Biology, Faculty of Life Sciences, University of Benin, Benin City, Edo State Nigeria; 6 Parasitology Institute (Zoology), University of Hohenheim, Stuttgart, Germany; 7 Justus-Liebig-University Giessen, Institute for Parasitology, Biomedical Research Centre Seltersberg (BFS), Schubertstr 81, D-35392 Giessen, Germany; 8 Experimental Pneumology, Research Center Borstel, Airway Research Center North, Member of the German Center for Lung Research (DZL), Parkallee 22, D-23845 Borstel, Germany

Correction to: *Scientific Reports* 10.1038/s41598-020-59892-6, published online 25 February 2020

The original version of this Article contained errors. Joseph E. Igetei and Michael J. Doenhoff were incorrectly affiliated with ‘Department of Medical Parasitology, Faculty of Medicine, Alexandria University, Alexandria, Egypt.’

In addition, an affiliation was omitted for the author Sarah Richter, which is now listed as Affiliation 6.

The correct author affiliations are listed below:

Affiliation 1:

School of Life Sciences, University Park, University of Nottingham, Nottinghamshire, NG7 2RD, UK.

Marwa H. El-Faham, Joseph E. Igetei, Michael J. Doenhoff & Sarah Richter

Affiliation 2:

Department of Medical Parasitology, Faculty of Medicine, Alexandria University, Alexandria, Egypt.

Marwa H. El-Faham

Affiliation 3:

School of Pharmacy, Division of Molecular Therapeutics and Formulation, University of Nottingham, Nottingham, NG7 2RD, UK.

Fatou Gai & Franco H. Falcone

Affiliation 4:

Ministry of Health & Social Welfare, National Public Health Laboratories, Banjul, The Gambia.

Fatou Gai

Affiliation 5:

Department of Animal and Environmental Biology, Faculty of Life Sciences, University of Benin, Benin City, Edo State, Nigeria.

Joseph E. Igetei

Affiliation 6:

Parasitology Institute (Zoology), University of Hohenheim, Stuttgart, Germany.

Sarah Richter

Affiliation 7:

Justus-Liebig-University Giessen, Institute for Parasitology, Biomedical Research Centre Seltersberg (BFS), Schubertstr 81, D-35392, Giessen, Germany.

Franco H. Falcone

Affiliation 8:

Experimental Pneumology, Research Center Borstel, Airway Research Center North, Member of the German Center for Lung Research (DZL), Parkallee 22, D-23845, Borstel, Germany.

Gabi Schramm

These errors have now been corrected in the PDF and HTML versions of the Article and in the accompanying Supplementary Information file.

