# Supplementary material for: Antigenic cross-reactivity between Schistosoma mansoni and allergenic invertebrates putatively due to shared glycanic epitopes
Source: Sci Rep. 2020 Feb 25;10:3350. doi: 10.1038/s41598-020-59892-6 (PMC7042331; doi:10.1038/s41598-020-59892-6)
Supplement: Supplementary file 1 — supplementary materials. [file 41598_2020_59892_MOESM1_ESM.docx]

**Supplementary Figures (S1-S8) and Supplementary Tables (S1-S10)**

**Antigenic cross-reactivity between *Schistosoma mansoni* and allergenic invertebrates putatively due to shared glycanic epitopes**

**Marwa H. El-Faham^$1^, Fatou Gai^$2,3^*, Joseph E. Igetei^4,5^, Sarah Richter^6^, Franco H. Falcone^2^, Gabi Schramm^7^, and Michael J. Doenhoff^4^**

*^$^Indicates equal contributions.*

*^1^Department of Medical Parasitology, Faculty of Medicine, Alexandria University, Egypt.*

*^2^School of Pharmacy, Division of Molecular Therapeutics and Formulation, University of Nottingham, Nottingham, UK NG7 2RD.*

*^3^Ministry of Health & Social Welfare, National Public Health Laboratories, Banjul, The Gambia.*

*^4^School of Life Sciences, University Park, University of Nottingham, Nottinghamshire, UK NG7 2RD.*

*^5^Department of Animal and Environmental Biology, Faculty of Life Sciences, University of Benin, Benin City, Edo State, Nigeria.*

*^6^Parasitology Institute (Zoology), University of Hohenheim, Stuttgart, Germany.*

*^7^Experimental Pneumology, Research Center Borstel, Airway Research Center North, Member of the German Center for Lung Research (DZL), Parkallee 22, D-23845 Borstel, Germany*

***Correspondence:**

Fatou Gai: Email: paxfg3@nottingham.ac.uk

**
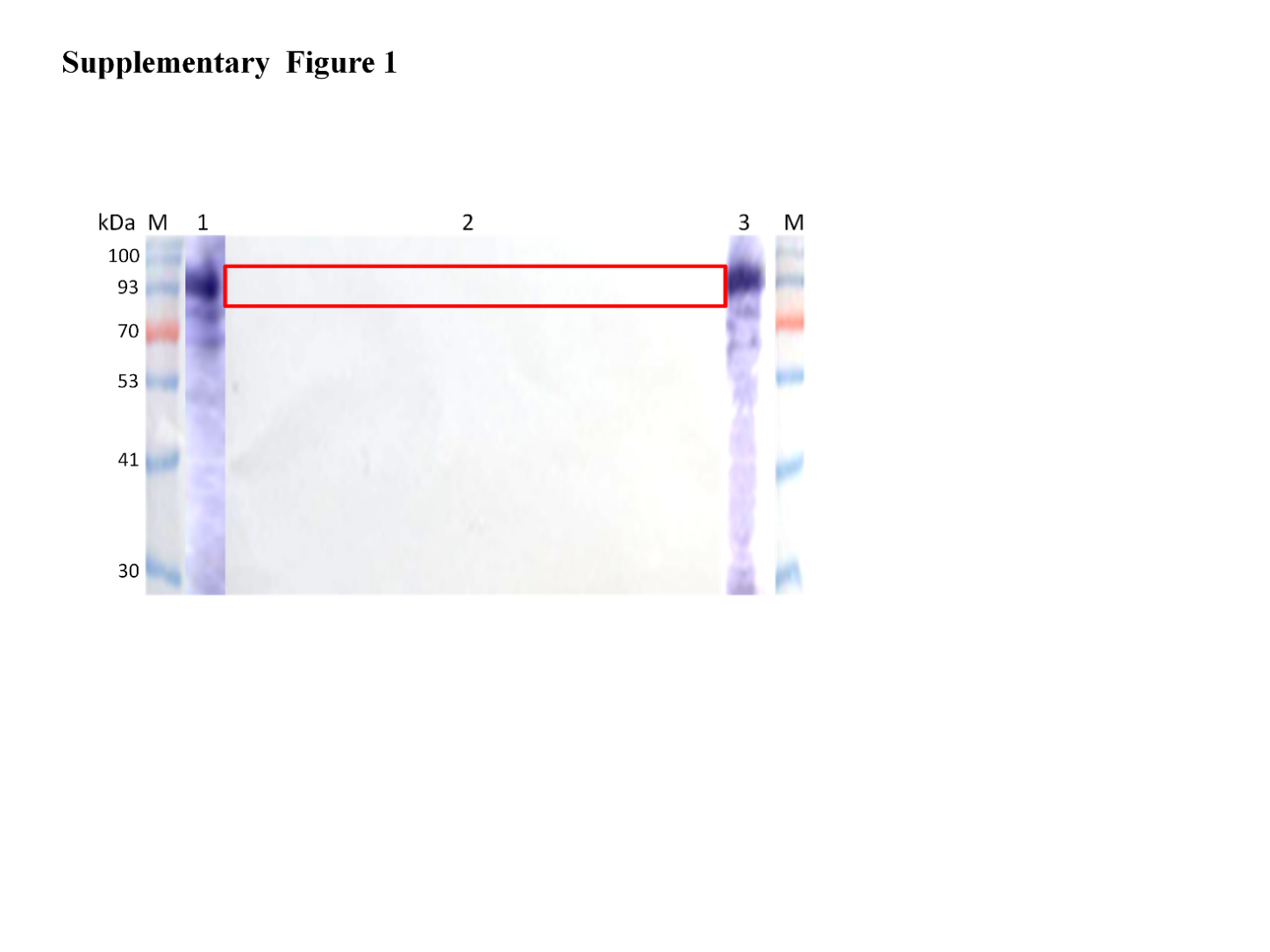
**

**Supplementary Figure S1. Western blot of a *D. farinae* extract probed with rabbit BR84 anti-SmSEA antiserum for acid-elution of antibodies reacting against a >90 kDa *D. farinae* antigen.**

200 µl *D. farinae* extract containing 660 µg protein was loaded into a single, wide lane in a 12% SDS-PAGE gel and electro-transferred to a nitrocellulose membrane. The membrane was incubated with rabbit anti-SmSEA antiserum BR84. Lanes 1 and 3 were then removed, washed and incubated with horse-radish peroxidase-conjugated goat anti-rabbit IgG antibodies and chromogenically stained. The red-outlined box indicates the position of the undeveloped lane 2 which was used for acid-elution of antibodies against the >90 kDa antigen by incubation with glycine/HCl buffer (pH 2.8).


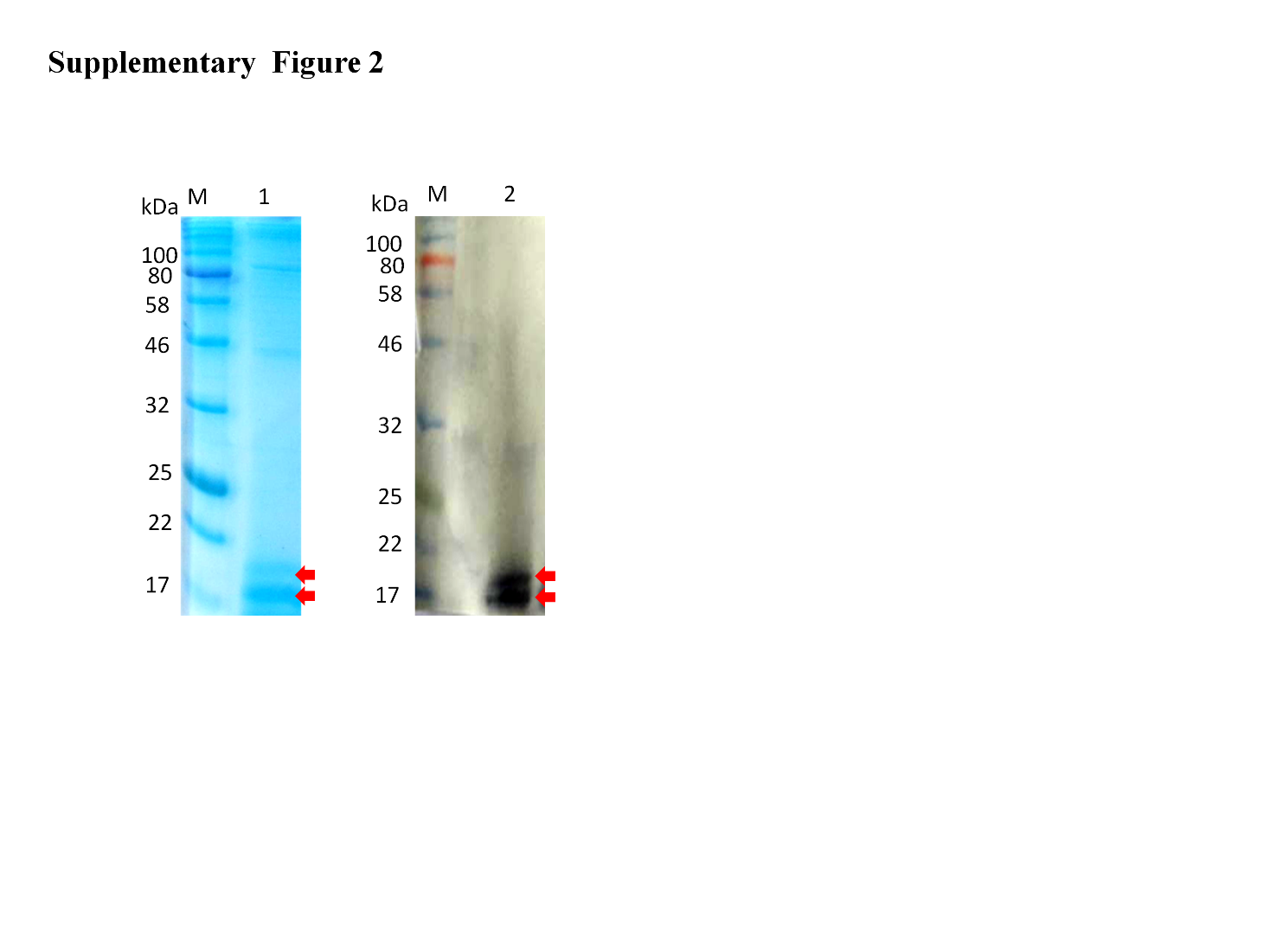


**Supplementary Figure S2. Coomassie blue-stained 12% acrylamide SDS-PAGE (lane 1) and Western immunoblot (lane 2) of purified bee venom 17-19 kDa proteins (25 µg/lane).**

Lane 1 was used to purify the ~17 and ~19 kDa bee venom protein bands (arrowed), which were analysed by TMS and both identified as PLA2. Lane 2 immunoblot was probed with a rabbit anti-PLA2 antiserum.


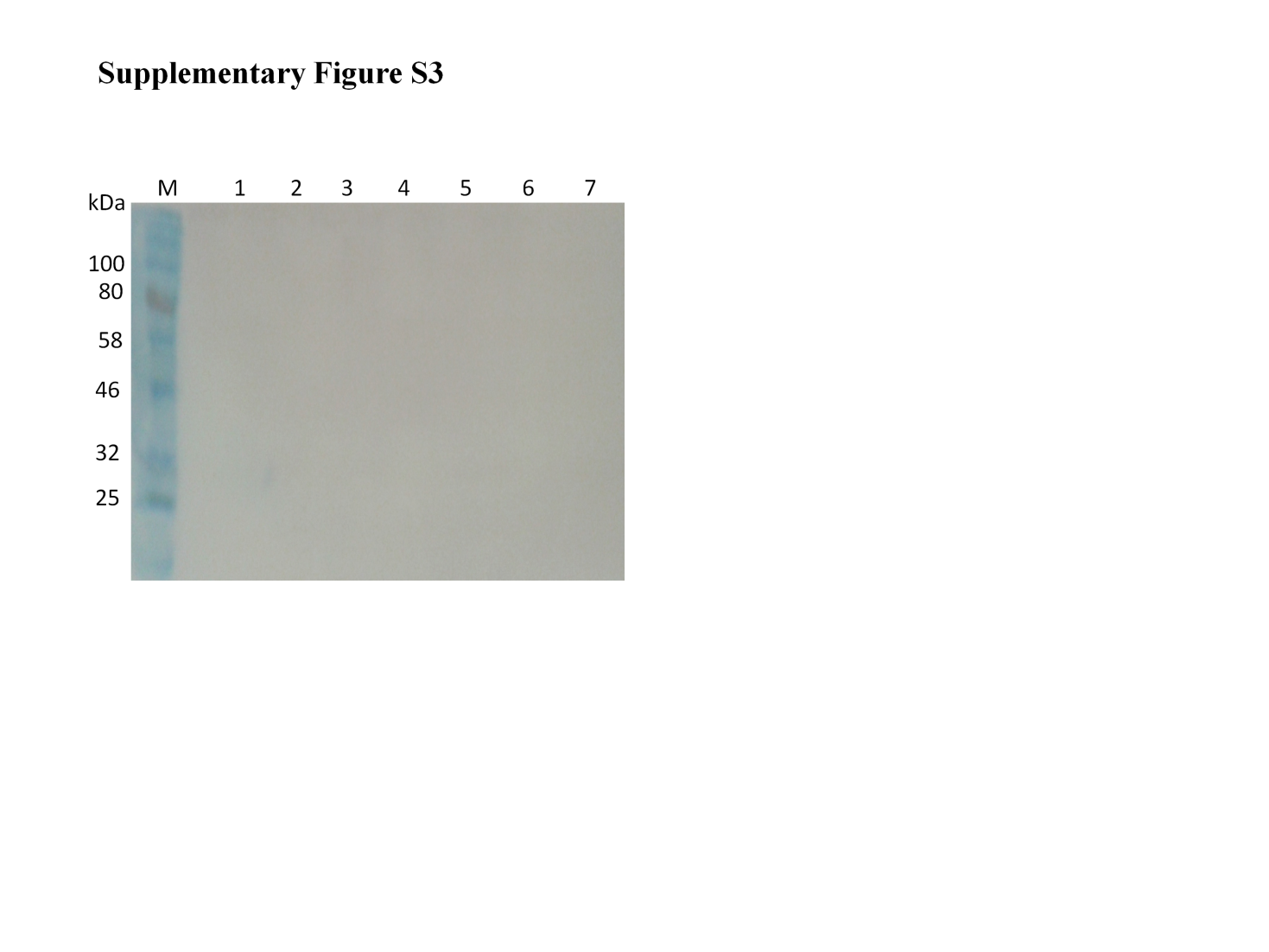


**Supplementary Figure S3. Western immunoblot of *S. mansoni* extracts and crude allergenic plant extracts probed with normal rabbit sera.**

1, *S. mansoni* SEA (20 µg/lane); 2, peanut (167 µg/lane); 3, tomato (188 µg/lane); 4, kiwi fruit (94 µg/lane); 5, latex (157.5 µg/lane); 6, *S. mansoni* worm homogenate (20 µg/lane); 7, *S. mansoni* cercariae homogenate (20 µg/lane). M = protein molecular size marker.


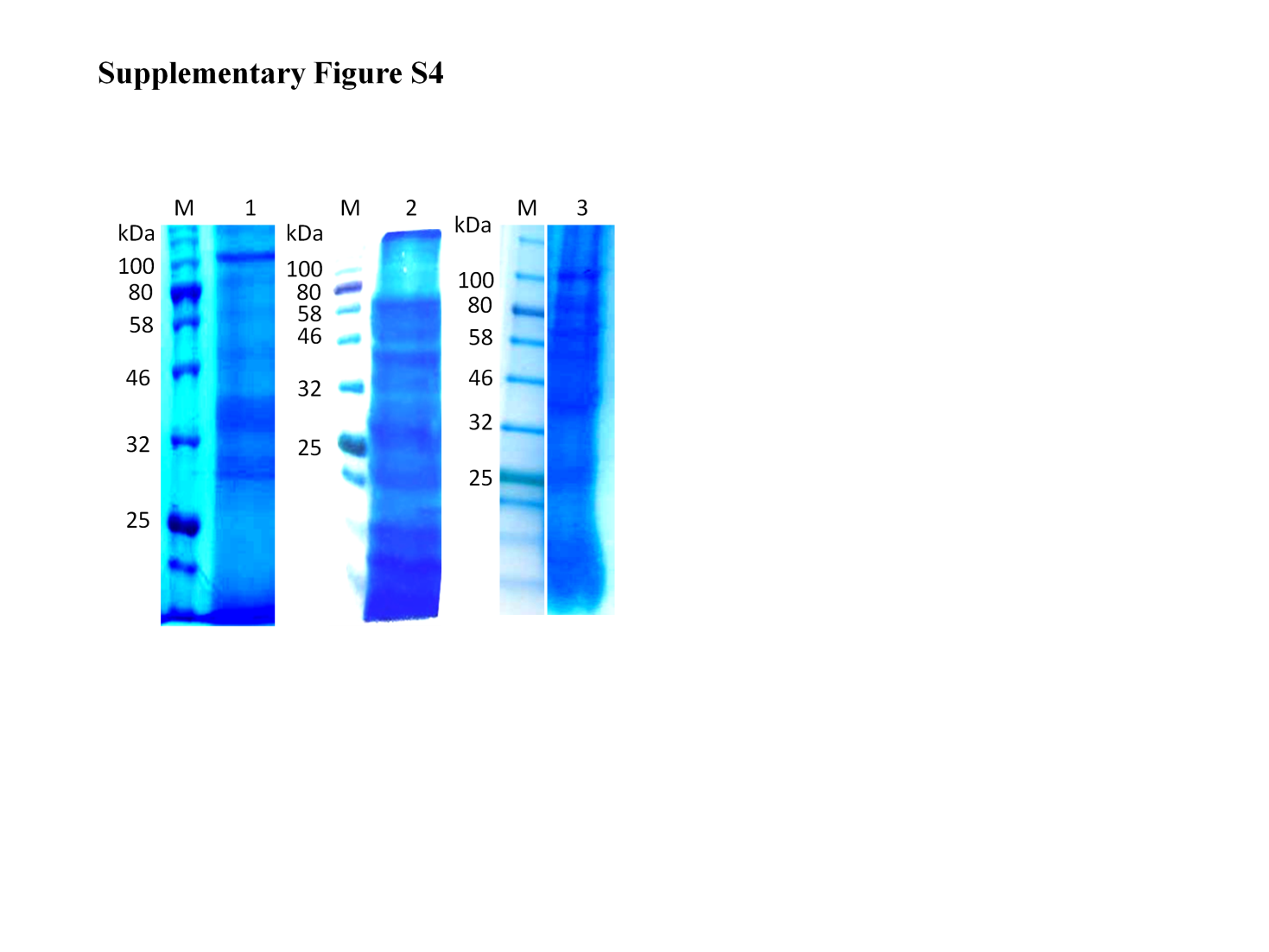


**Supplementary Figure S4. Coomassie blue-stained SDS-PAGE gel of *S. mansoni* extracts**

1, *S. mansoni* SEA (20 µg/lane); 2, *S. mansoni* worm homogenate (20 µg/lane); 3, *S. mansoni* cercariae homogenate (20 µg/lane). M = protein molecular size marker.

**
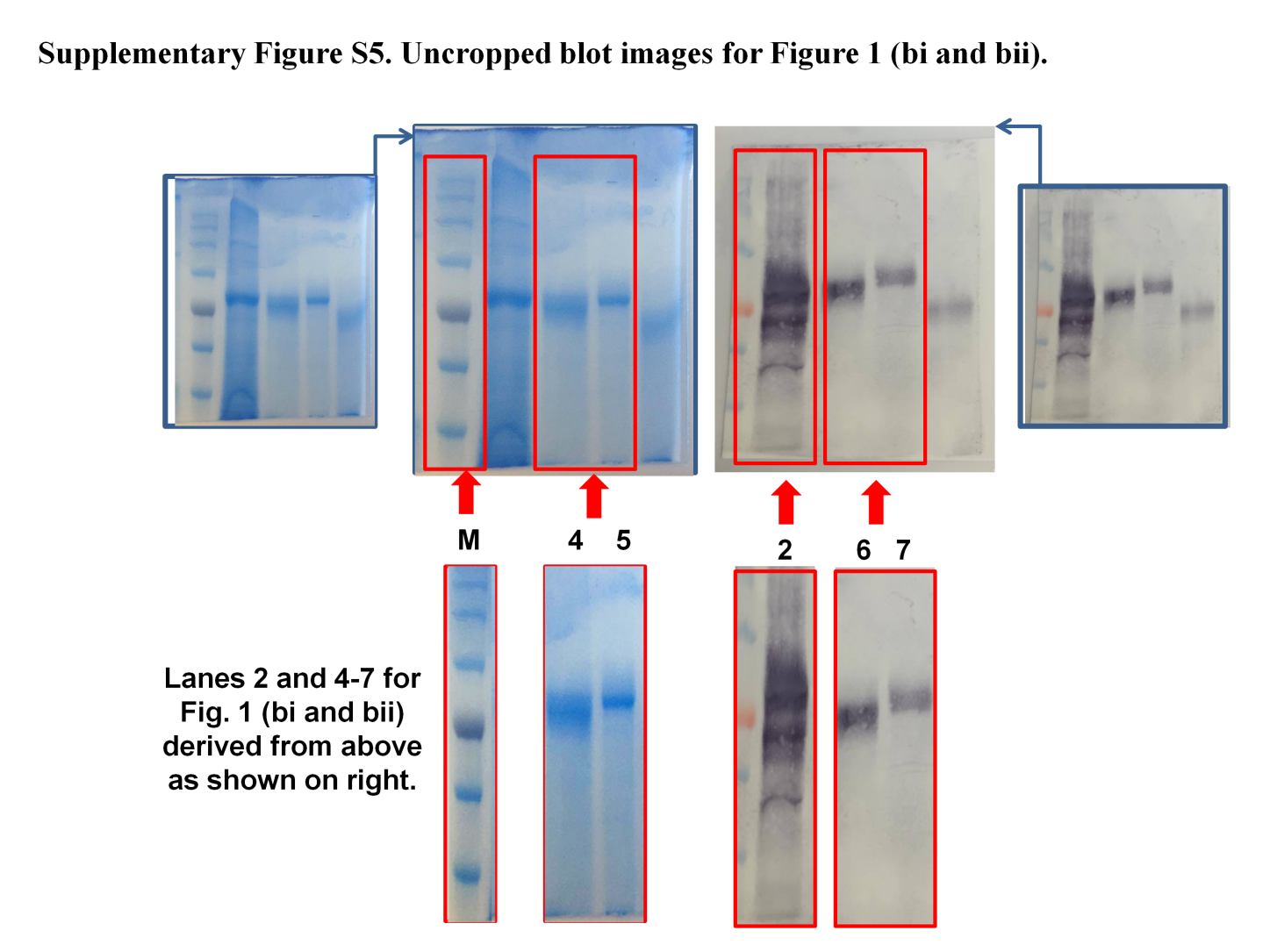
**

**Supplementary Figure S5. Uncropped blot images for Figure 1 (bi and bii).**

**
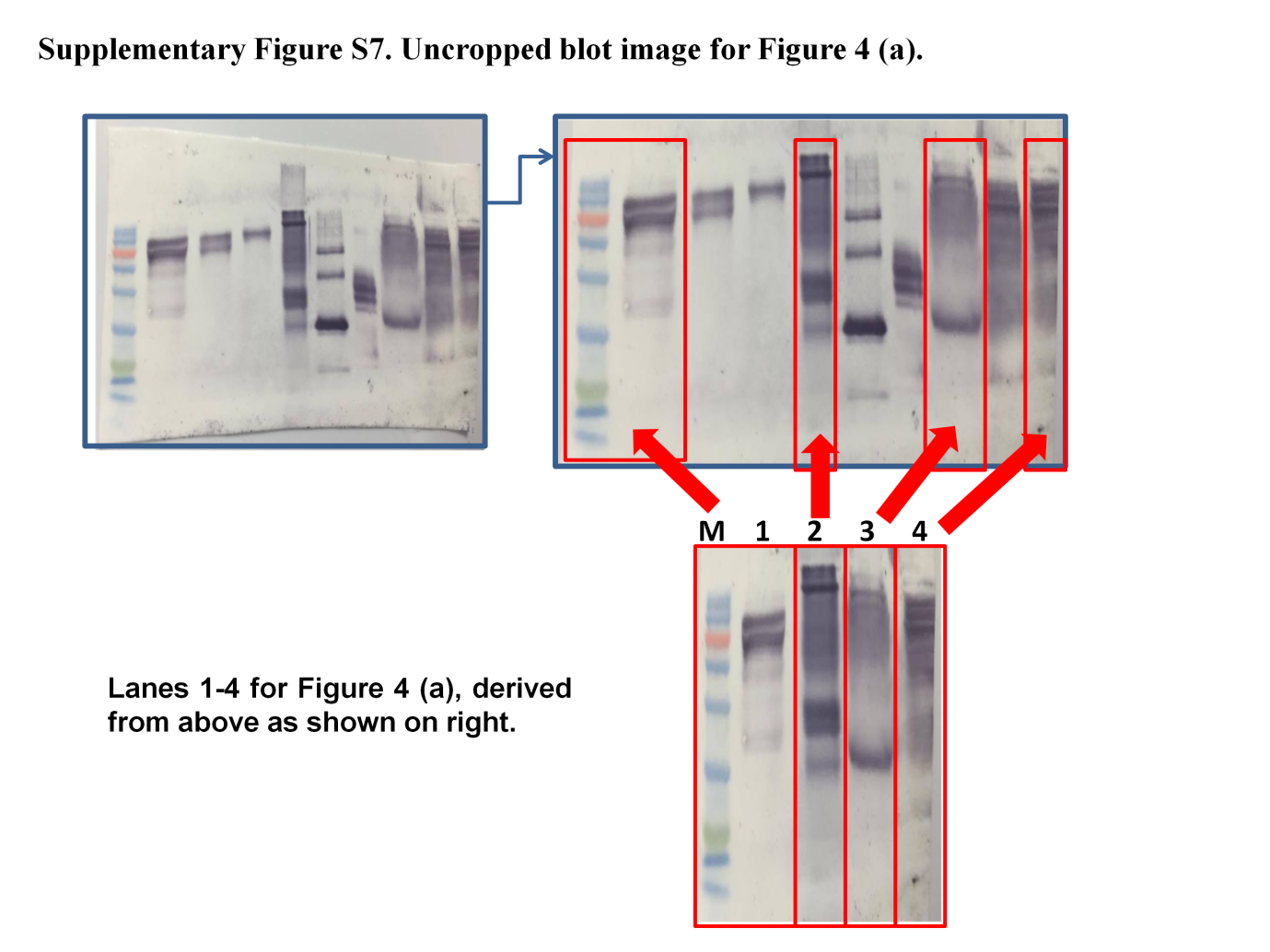
**

**Supplementary Figure S6. Uncropped blot images for Figure 4 (a).**

**
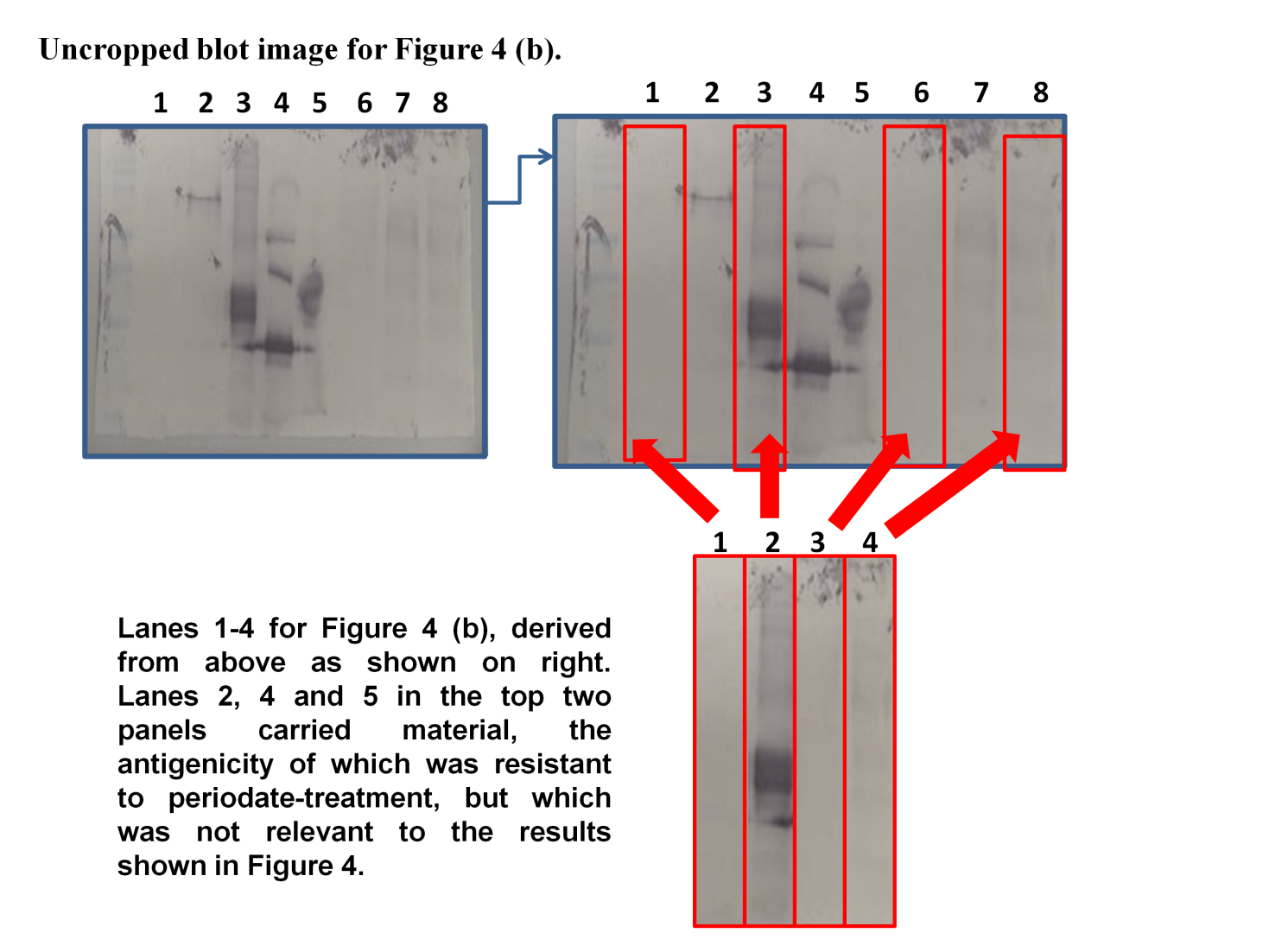
**

**Supplementary Figure S7. Uncropped blot images for Figure 4 (b).**

**
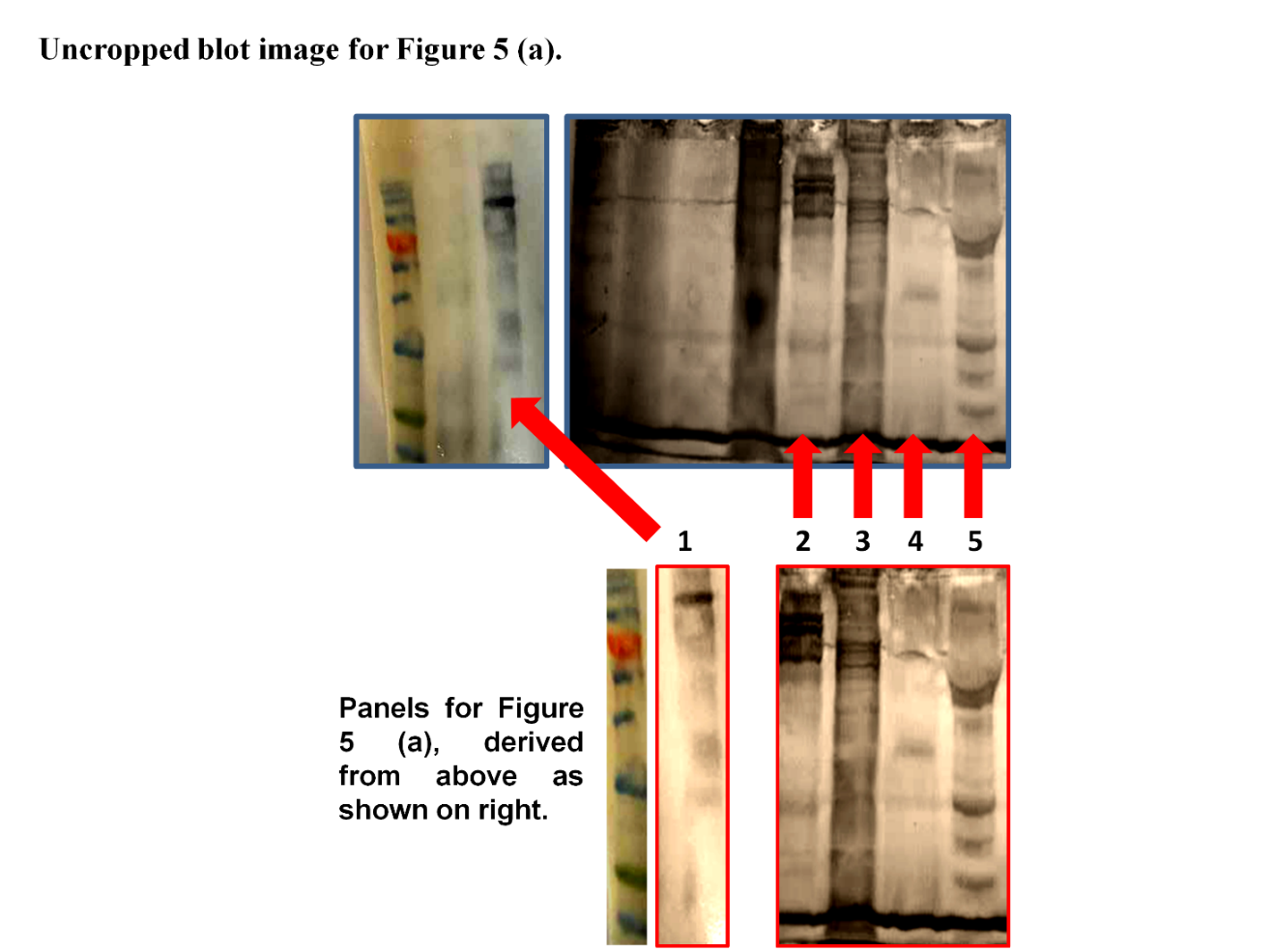
**

**Supplementary Figure S8. Uncropped blot images for Figure 5 (a).**

**Supplementary Table S1. MASCOT search output of NCBInr with the tandem MS data from the purified >90 kDa *D. farinae* gel band.**

| **gi:** 5815436 **98 kDa HDM allergen [*Dermatophagoides farinae*]**  **Mass:** 63882 Da  **Score:** 175 **Matches:** 11 (4) **Sequences:** 10 (4) **emPAI**^a^**:** 0.35 | | | | | | | | |
| --- | --- | --- | --- | --- | --- | --- | --- | --- |
| **Peptide match** | | | **Score** | | **Expect** | **Rank** | | **Unique** |
| R.IVCYVGTWSVYHK.V | | | 81 | | 1.2e-06 | 1 | | U |
| K.VDPYTIEDIDPFK.C | | | 101 | | 8.3e-07 | 1 | | U |
| K.YSDMAANPTYR.Q | | | 57 | | 0.0016 | 1 | | U |
| K.IDKQNYLALVR.E | | | 68 | | 0.00056 | 1 | | U |
| K.QNYLALVR.E | | | 57 | | 0.58 | 1 | | U |
| R.ELKDAFEPHGYLLTAAVSPGK.D | | | 9 | | 2.6 | 4 | | U |
| K.DAFEPHGYLLTAAVSPGKDK.I | | | 2 | | 0.85 | 1 | | U |
| K.LVMGVPFYGR.A | | | 21 | | 0.3 | 1 | | U |
| K.LVMGVPFYGR.A + Oxidation (M) | | | 26 | | 4.3 | 3 | | U |
| R.AWSIEDR.S | | | 36 | | 0.39 | 1 | | U |
| K.EGDIPHPTNIHK.Y | | | 22 | | 0.077 | 1 | | U |
| **Percentage sequence coverage:** 18 %. Matched peptides shown in bold and underlined. | | | | | | | | |
| 1 MKTIYAILSI | MACIGLMNAS | IKRDHNDYSK | | NPMR**IVCYVG** | | | **TWSVYHKVDP** | |
| 51 **YTIEDIDPFK** | CTHLMYGFAK | IDEYKYTIQV | | FDPYQDDNHN | | | SWEKRGYERF | |
| 101 NNLRLKNPEL | TTMISLGGWY | EGSEK**YSDMA** | | **ANPTYR**QQFI | | | QSVLDFLQEY | |
| 151 KFDGLDLDWE | YPGSRLGNPK | **IDKQNYLALV** | | **RELKDAFEPH** | | | **GYLLTAAVSP** | |
| 201 **GKDK**IDRAYD | IKELNKLFDW | MNVMTYDYHG | | GWENFYGHNA | | | PLYKRPDETD | |
| 251 ELHTYFNVNY | TMHYYLNNGA | TRDK**LVMGVP** | | **FYGRAWSIED** | | | **R**SKLKLGDPA | |
| 301 KGMSPPGFIS | GEEGVLSYIE | LCQLFQKEEW | | HIQYDEYYNA | | | PYGYNDKIWV | |
| 351 GYDDLASISC | KLAFLKELGV | SGVMVWSLEN | | DDFKGHCGPK | | | NPLLNKVHNM | |
| 401 INGDEKNSFE | CILGPSTTTP | TPTTTPTTPT | | TTPTTPSPTT | | | PTTTPSPTTP | |
| 451 TTTPSPTTPT | TTPSPTTPTP | TTPTPAPTTS | | TPSPTTTEHT | | | SETPKYTTYV | |
| 501 DGHLIKCYK**E** | **GDIPHPTNIH** | **K**YLVCEFVNG | | GWWVHIMPCP | | | PGTIWCQEKL | |
| 551 TCIGE |  |  | |  | | |  | |

^a^emPAI, the exponentially modified protein abundance index.

**Supplementary Table S2. MASCOT search output of NCBInr with the tandem MS data from the purified ~85 kDa cockroach gel band.**

| **gi:** 284518363 **Allergen Cr-PI; AltName: Allergen Per a 3 [*Periplaneta americana*]**  **Mass:** 82283 Da  **Score:** 884 **Matches:** 25 (25) **Sequences:** 22 (22) **emPAI**^a^**:** 3.01 | | | | | | | |
| --- | --- | --- | --- | --- | --- | --- | --- |
| **Peptide match** | | | **Score** | **Expect** | **Rank** | | **Unique** |
| R.VNEGMFMYSFSIAVFHR.D | | | 55 | 3.8e-05 | 1 | |  |
| K.NQDQLLAYFTSDVNLNAFNTYYR.Y | | | 136 | 1.7e-12 | 1 | | U |
| R.GEQFYYTYK.Q | | | 52 | 0.0048 | 1 | |  |
| R.GEQFYYTYKQIYAR.Y | | | 66 | 3e-05 | 1 | |  |
| R.LSNDLPDVYPFYYSKPVK.S | | | 58 | 0.00018 | 1 | |  |
| R.YHNGEEMPVRPSNLYVTNFDLYYIADIK.N + oxidation | | | 60 | 3.3e-06 | 1 | | U |
| K.MGLAPSALEHPETVLR.D | | | 109 | 3.9e-09 | 1 | | U |
| K.MGLAPSALEHPETVLR.D + oxidation (M) | | | 109 | 1.4e-08 | 1 | | U |
| R.DPAFYQLWK.R+ oxidation | | | 43 | 9.2e-05 | 1 | | U |
| R.DPAFYQLWK.R | | | 52 | 0.02 | 1 | |  |
| R.YTHDELAFEGVK.V | | | 52 | 0.0016 | 1 | |  |
| K.LYTYFEQYDVSLDMSVYVNK.V | | | 63 | 6.5e-06 | 1 | | U |
| K.VDQIPNVDVHAR.Q | | | 44 | 0.015 | 1 | | U |
| R.LNHKPFTYNIEVSSDK.A | | | 115 | 1.5e-07 | 1 | |  |
| R.NSHDSNIVAPER.D | | | 89 | 9.1e-07 | 1 | | U |
| R.NSHDSNIVAPERDSYR.T | | | 59 | 3.7e-05 | 1 | | U |
| K.GHNYCGYPENLLIPK.G | | | 53 | 0.0013 | 1 | |  |
| K.GHNYCGYPENLLIPK.G | | | 49 | 0.0016 | 1 | |  |
| K.KGGQAYTFYVIVTPYVK.Q | | | 61 | 0.0003 | 1 | |  |
| K.GGQAYTFYVIVTPYVK.Q | | | 86 | 4.7e-07 | 1 | |  |
| K.QDEHDFEPYNYK.A | | | 71 | 2e-05 | 1 | |  |
| K.AFSYCGVGSNR.K | | | 84 | 6.2e-06 | 1 | | U |
| K.AFSYCGVGSNRK.Y | | | 37 | 0.028 | 1 | | U |
| K.IYSNDFYTPNMYFK.D | | | 35 | 0.0022 | 1 | |  |
| K.IYSNDFYTPNMYFK.D + oxidation (M) | | | 71 | 4.4e-06 | 1 | |  |
| **Percentage sequence coverage:** 39%. Matched peptides shown in bold and underlined. | | | | | | | |
| 1 MKTALVFAAV | VALVACAAFP | AHKDYKQLAD | | KQFLAKQRDV | | LRLFHRVHQH | |
| 51 NILNDQVEVG | NTYDIEANIG | NYKYPRVVKQ | | FMAYFKKGML | | PRGEPFSVYF | |
| 101 EKHREQAIML | YNLFYFANDY | DTFYKTACWA | | RDR**VNEGMFM** | | **YSFSIAVFHR** | |
| 151 DDMQGVMLPP | PYEVYPYLFV | DHDVIHMAQK | | YWMKNAGSNE | | HHSYVIPVNF | |
| 201 TLK**NQDQLLA** | **YFTSDVNLNA** | **FNTYYR**YYYP | | SWYNTTLYGH | | TIDRR**GEQFY** | |
| 251 **YTYKQIYAR**Y | FLER**LSNDLP** | **DVYPFYYSKP** | | **VK**SAYNPNLR | | **YHNGEEMPVR** | |
| 301 **PSNLYVTNFD** | **LYYIADIK**NY | EKRVEDAIDF | | GYVFDEHVKP | | HSLYHDVHGM | |
| 351 EYVADMIEGN | MDSPNFYFYG | SIYHMYHSMI | | GHIVDPYHK**M** | | **GLAPSALEHP** | |
| 401 **TVLRDPAFY** | **QLWK**RVDHLF | QKYKNRLPR**Y** | | **THDELAFEGV** | | **K**VENVDVGK**L** | |
| 451 **YTYFEQYDVS** | **LDMSVYVNKV** | **DQIPNVDVHA** | | **R**QYR**LNHKPF** | | **TYNIEVSSDK** | |
| 501 AQDVYVRVFL | GPKYDYLGRE | YDLNDRRHYF | | VEMDRFPHHV | | EAGKTVIER**N** | |
| 551 **SHDSNIVAPE** | **RDSYR**TFYKK | VQEAYEGKSQ | | YYVDK**GHNYC** | | **GYPENLLIPK** | |
| 601 GK**KGGQAYTF** | **YVIVTPYVKQ** | **DEHDFEPYNY** | | **KAFSYCGVGS** | | **NRK**YPDNMPL | |
| 651 GYPFDRK**IYS** | **NDFYTPNMYF** | **K**DVIIFHKKY | | DEVGVQGH | |  | |

^a^emPAI, the exponentially modified protein abundance index.

**Supplementary Table S3. MASCOT search output of NCBInr with the tandem MS data from the purified ~88 kDa cockroach gel band.**

| **gi:** 284518363 **Allergen Cr-PI; AltName: Allergen Per a 3 [*Periplaneta americana*]**  **Mass:** 82283 Da  **Score:** 239 **Matches:** 7 (7) **Sequences:** 7 (7) **emPAI**^a^**:** 0.50 | | | | | | | | |
| --- | --- | --- | --- | --- | --- | --- | --- | --- |
| **Peptide match** | | | **Score** | **Expect** | | **Rank** | | **Unique** |
| K.NQDQLLAYFTSDVNLNAFNTYYR.Y | | | 113 | 5.9e-10 | | 1 | | U |
| R.GEQFYYTYK.Q | | | 47 | 0.0038 | | 1 | |  |
| K.MGLAPSALEHPETVLR.D | | | 96 | 3.8e-07 | | 1 | | U |
| R.DPAFYQLWK.R | | | 62 | 0.001 | | 1 | |  |
| R.YTHDELAFEGVK.V | | | 85 | 3e-05 | | 1 | |  |
| K.VENVDVGK.L | | | 57 | 0.043 | | 1 | |  |
| R.NSHDSNIVAPER.D | | | 42 | 0.043 | | 1 | | U |
| **Percentage sequence coverage:** 12%. Matched peptides shown in bold and underlined. | | | | | | | | |
| 1 MKTALVFAAV | VALVACAAFP | AHKDYKQLAD | | | KQFLAKQRDV | | LRLFHRVHQH | |
| 51 NILNDQVEVG | NTYDIEANIG | NYKYPRVVKQ | | | FMAYFKKGML | | PRGEPFSVYF | |
| 101 EKHREQAIML | YNLFYFANDY | DTFYKTACWA | | | RDRVNEGMFM | | YSFSIAVFHR | |
| 151 DDMQGVMLPP | PYEVYPYLFV | DHDVIHMAQK | | | YWMKNAGSNE | | HHSYVIPVNF | |
| 201 TLK**NQDQLLA** | **YFTSDVNLNA** | **FNTYYR**YYYP | | | SWYNTTLYGH | | TIDRR**GEQFY** | |
| 251 **YTYK**QIYARY | LYYIADIKNY | EKRVEDAIDF | | | GYVFDEHVKP | | HSLYHDVHGM | |
| 301 PSNLYVTNFD | LYYIADIKNY | EKRVEDAIDF | | | GYVFDEHVKP | | HSLYHDVHGM | |
| 351 EYVADMIEGN | MDSPNFYFYG | SIYHMYHSMI | | | GHIVDPYHK**M** | | **GLAPSALEHP** | |
| 401 **ETVLRDPAFY** | **QLWK**RVDHLF | QKYKNRLPR**Y** | | | **THDELAFEGV** | | **KVENVDVGK**L | |
| 451 YTYFEQYDVS | LDMSVYVNKV | DQIPNVDVHA | | | RQYRLNHKPF | | TYNIEVSSDK | |
| 501 AQDVYVRVFL | GPKYDYLGRE | YDLNDRRHYF | | | VEMDRFPHHV | | EAGKTVIER**N** | |
| 551 **SHDSNIVAPE** | **R**DSYRTFYKK | VQEAYEGKSQ | | | YYVDKGHNYC | | GYPENLLIPK | |
| 601 GKKGGQAYTF | YVIVTPYVKQ | DEHDFEPYNY | | | KAFSYCGVGS | | NRKYPDNMPL | |
| 651 GYPFDRKIYS | NDFYTPNMYF | KDVIIFHKKY | | | DEVGVQGH | |  | |

^a^emPAI, the exponentially modified protein abundance index.

**Supplementary Table S4. MASCOT search output of NCBInr with the tandem MS data from the purified ~19 kDa bee venom gel band.**

| **gi:** 5627  **phospholipase A-2 [*Apis mellifera*]**  **Mass:** 19045 Da  **Score:** 349 **Matches:** 12 (12) **Sequences:** 8 (8) **emPAI**^a^**:** 8.61 | | | | | | | |
| --- | --- | --- | --- | --- | --- | --- | --- |
| **Peptide match** | | | **Score** | **Expect** | **Rank** | | **Unique** |
| K.SSGPNELGR.F | | | 96 | 1.5e-06 | 1 | |  |
| K.HGLTNTASHTR.L | | | 68 | 3.1e-05 | 1 | | U |
| R.LSCDCDDKFYDCLK.N | | | 36 | 0.0014 | 1 | | U |
| R.LSCDCDDKFYDCLK.N | | | 67 | 4.5e-06 | 1 | | U |
| K.NSADTISSYFVGK.M | | | 94 | 1.5e-06 | 1 | | U |
| K.MYFNLIDTK.C | | | 50 | 0.00055 | 1 | | U |
| K.MYFNLIDTK.C + oxidation (M) | | | 74 | 1.8e-05 | 1 | | U |
| K.MYFNLIDTK.C + oxidation (M) | | | 54 | 0.00039 | 1 | | U |
| K.MYFNLIDTK.C + oxidation (M) | | | 48 | 0.0016 | 1 | | U |
| K.LEHPVTGCGER.T | | | 59 | 0.00085 | 1 | |  |
| K.VYQWFDLR.K | | | 53 | 0.0043 | 1 | |  |
| K.VYQWFDLRK.Y | | | 40 | 0.0051 | 1 | |  |
| **Percentage sequence coverage:** 46%. Matched peptides shown in bold and underlined. | | | | | | | |
| 1 GSLFLLLLST | SHGWQIRDRI | GDNELEERII | | YPGTLWCGHG | | NK**SSGPNELG** | |
| 51 **R**FKHTDACCR | THDMCPDVMS | AGESK**HGLTN** | | **TASHTRLSCD** | | **CDDKFYDCLK** | |
| 101 **NSADTISSYF** | **VGKMYFNLID** | **TK**CYK**LEHPV** | | **TGCGER**TEGR | | CLHYTVDKSK | |
| 151 PK**VYQWFDLR** | **K**Y |  | |  | |  | |

^a^emPAI, the exponentially modified protein abundance index.

**Supplementary Table S5. MASCOT search output of NCBInr with the tandem MS data from the purified ~17 kDa bee venom gel band.**

| **gi:** 5627  **phospholipase A-2 [*Apis mellifera*]**  **Mass:** 19045 Da  **Score:** 558 **Matches:** 20 (20) **Sequences:** 11 (11) **emPAI**^a^**:** 26.28 | | | | | | | |
| --- | --- | --- | --- | --- | --- | --- | --- |
| **Peptide match** | | | **Score** | **Expect** | **Rank** | | **Unique** |
| R.IIYPGTLWCGHGNK.S | | | 67 | 3.7e-06 | 1 | |  |
| K.SSGPNELGR.F | | | 76 | 6.2e-05 | 1 | |  |
| R.THDMCPDVMSAGESK.H + oxidation (M) | | | 44 | 9.5e-05 | 1 | | U |
| R.THDMCPDVMSAGESK.H + 2 oxidation (M) | | | 63 | 4.5e-06 | 1 | | U |
| K.HGLTNTASHTR.L | | | 85 | 5.9e-07 | 1 | | U |
| R.LSCDCDDKFYDCLK.N | | | 47 | 7.1e-05 | 1 | | U |
| K.NSADTISSYFVGK.M | | | 91 | 2.5e-06 | 1 | | U |
| K.NSADTISSYFVGK.M | | | 43 | 0.0039 | 1 | | U |
| K.NSADTISSYFVGK.M | | | 82 | 2.1e-05 | 1 | | U |
| K.NSADTISSYFVGK.M | | | 91 | 9e-07 | 1 | | U |
| K.NSADTISSYFVGK.M | | | 88 | 3.5e-07 | 1 | | U |
| K.MYFNLIDTK.C | | | 53 | 0.015 | 1 | | U |
| K.MYFNLIDTK.C + oxidation (M) | | | 50 | 0.0021 | 1 | | U |
| K.MYFNLIDTK.C + oxidation (M) | | | 73 | 3e-05 | 1 | | U |
| K.LEHPVTGCGERTK.CY | | | 35 | 0.0022 | 1 | |  |
| K.LEHPVTGCGER.T | | | 60 | 0.0043 | 1 | |  |
| R.CLHYTVDK.S | | | 36 | 0.03 | 1 | |  |
| K.VYQWFDLR.K | | | 56 | 0.0046 | 1 | |  |
| K.VYQWFDLR.K | | | 57 | 0.0045 | 1 | |  |
| K.VYQWFDLR.K | | | 56 | 0.0051 | 1 | |  |
| **Percentage sequence coverage:** 70%. Matched peptides shown in bold and underlined. | | | | | | | |
| 1 GSLFLLLLST | SHGWQIRDRI | GDNELEER**II** | | **YPGTLWCGHG** | | **NKSSGPNELG** | |
| **51 R**FKHTDACCR | **THDMCPDVMS** | **AGESKHGLTN** | | **TASHTRLSCD** | | **CDDKFYDCLK** | |
| **101 NSADTISSYF** | **VGKMYFNLID** | **TKCYKLEHPV** | | **TGCGER**TEGR | | **CLHYTVDK**SK | |
| 151 PK**VYQWFDLR** | KY |  | |  | |  | |

^a^emPAI, the exponentially modified protein abundance index.

**Supplementary Table S6: Potential N-linked glycosylation sites for Der f 15.**

>Der f15 Length = 555

MKTIYAILSIMACIGLM**N**ASIKRDHNDYSKNPMRIVCYVGTWSVYHKVDPYTIEDIDPFKCTHLMYGFAKIDEYKYTIQVFDPYQDDNHNSWEKRGYERFNNLRLKNPELTTMISLGGWYEGSEKYSDMAA**N**PTYRQQFIQSVLDFLQEYKFDGLDLDWEYPGSRLGNPKIDKQNYLALVRELKDAFEPHGYLLTAAVSPGKDKIDRAYDIKELNKLFDWMNVMTYDYHGGWENFYGHNAPLYKRPDETDELHTYFNV**N**YTMHYYLNNGATRDKLVMGVPFYGRAWSIEDRSKLKLGDPAKGMSPPGFISGEEGVLSYIELCQLFQKEEWHIQYDEYYNAPYGYNDKIWVGYDDLASISCKLAFLKELGVSGVMVWSLENDDFKGHCGPKNPLLNKVHNMINGDEKNSFECILGPSTTTPTPTTTPTTPTTTPTTPSPTTPTTTPSPTTPTTTPSPTTPTTTPSPTTPTPTTPTPAPTTSTPSPTTTEHTSETPKYTTYVDGHLIKCYKEGDIPHPTNIHKYLVCEFVNGGWWVHIMPCPPGTIWCQEKLTCIGE

| **Position** | **Residue** | **Score^*^** | **Jury agreement^∞^** | **N-Glyc result^$^** |
| --- | --- | --- | --- | --- |
| **18** | **N**AS | 0.6181 | (9/9) | ++ |
| **132** | **N**PT | 0.6447 | (8/9) | + |
| **259** | **N**YT | 0.7652 | (9/9) | +++ |

**^*^**The score is the averaged output of nine neural networks.

**^∞^**The jury agreement column indicates how many of the nine networks support the prediction.

**^$^**+ Score > 0.5; ++ Score > 0.5 and Jury agreement (9/9) or Score >0.75; +++ Score > 0.75 and Jury agreement (9/9) ; ++++ Score > 0.90 and Jury agreement (9/9).

**Supplementary Table S7: Potential O-linked glycosylation sites for Der f 15.**

>Der f15 Length = 555

MKTIYAILSIMACIGLMNASIKRDHNDYSKNPMRIVCYVGTWSVYHKVDPYTIEDIDPFKCTHLMYGFAKIDEYKYTIQVFDPYQDDNHNSWEKRGYERFNNLRLKNPELTTMISLGGWYEGSEKYSDMAANPTYRQQFIQSVLDFLQEYKFDGLDLDWEYPGSRLGNPKIDKQNYLALVRELKDAFEPHGYLLTAAVSPGKDKIDRAYDIKELNKLFDWMNVMTYDYHGGWENFYGHNAPLYKRPDETDELHTYFNVNYTMHYYLNNGATRDKLVMGVPFYGRAWSIEDRSKLKLGDPAKGMSPPGFISGEEGVLSYIELCQLFQKEEWHIQYDEYYNAPYGYNDKIWVGYDDLA**S**ISCKLAFLKELGVSGVMVW**S**LENDDFKGHCGPKNPLLNKVHNMINGDEKN**S**FECILGP**STTT**P**T**P**TTT**P**TT**P**TTT**P**TT**P**S**P**TT**P**TTT**P**S**P**TT**P**TTT**P**S**P**TT**P**TTT**P**S**P**TT**P**T**P**TT**P**T**PAP**TTST**P**S**P**TTT**EH**TS**E**T**PKY**TT**YVDGHLIKCYKEGDIPHPTNIHKYLVCEFVNGGWWVHIMPCPPGTIWCQEKLTCIGE

| **Position** | **Residue** | **Score^*^** | **Prediction** |
| --- | --- | --- | --- |
| **304** | **S** | 0.746906 | O-Glycosylated  O-Glycosylated |
| **416** | **S** | 0.97124 |  |
| **417** | **T** | 0.978311 |  |
| **418** | **T** | 0.980472 |  |
| **419** | **T** | 0.98408 |  |
| **421** | **T** | 0.992813 |  |
| **423** | **T** | 0.998455 |  |
| **424** | **T** | 0.998705 |  |
| **425** | **T** | 0.997559 |  |
| **427** | **T** | 0.999381 |  |
| **428** | **T** | 0.99506 |  |
| **430** | **T** | 0.99884 |  |
| **431** | **T** | 0.998708 |  |
| **432** | **T** | 0.998308 |  |
| **434** | **T** | 0.998612 |  |
| **435** | **T** | 0.998758 |  |
| **437** | **S** | 0.992194 |  |
| **439** | **T** | 0.99598 |  |
| **440** | **T** | 0.988899 |  |
| **442** | **T** | 0.994358 |  |
| **443** | **T** | 0.995248 |  |
| **444** | **T** | 0.99669 |  |
| **446** | **S** | 0.978851 |  |
| **448** | **T** | 0.992388 |  |
| **449** | **T** | 0.978779 |  |
| **451** | **T** | 0.989288 |  |
| **452** | **T** | 0.992476 |  |
| **453** | **T** | 0.994422 |  |
| **455** | **S** | 0.967404 |  |
| **457** | **T** | 0.987643 |  |
| **458** | **T** | 0.968631 |  |
| **460** | **T** | 0.9806 |  |
| **461** | **T** | 0.98968 |  |
| **462** | **T** | 0.99234 |  |
| **464** | **S** | 0.987485 |  |
| **466** | **T** | 0.992013 |  |
| **467** | **T** | 0.983368 |  |
| **469** | **T** | 0.975937 |  |
| **471** | **T** | 0.994849 |  |
| **472** | **T** | 0.986307 |  |
| **474** | **T** | 0.989304 |  |
| **478** | **T** | 0.997451 |  |
| **479** | **T** | 0.988791 |  |
| **480** | **S** | 0.994586 |  |
| **481** | **T** | 0.992045 |  |
| **483** | **S** | 0.992686 |  |
| **485** | **T** | 0.979075 |  |
| **486** | **T** | 0.971932 |  |
| **487** | **T** | 0.989194 |  |
| **490** | **T** | 0.980241 |  |
| **491** | **S** | 0.988864 |  |
| **493** | **T** | 0.950198 |  |
| **497** | **T** | 0.931241 |  |
| **498** | **T** | 0.685178 |  |

**^*^** Residues with scores ≥ 0.5 were predicted as glycosylated.

**Supplementary Table S8: Potential N-linked glycosylation sites for Cr-PI/allergen Per a 3.**

>Per a3 Length = 688

MKTALVFAAVVALVACAAFPAHKDYKQLADKQFLAKQRDVLRLFHRVHQHNILNDQVEVGNTYDIEANIGNYKYPRVVKQFMAYFKKGMLPRGEPFSVYFEKHREQAIMLYNLFYFANDYDTFYKTACWARDRVNEGMFMYSFSIAVFHRDDMQGVMLPPPYEVYPYLFVDHDVIHMAQKYWMKNAGSNEHHSYVIPV**N**FTLKNQDQLLAYFTSDVNLNAFNTYYRYYYPSWY**N**TTLYGHTIDRRGEQFYYTYKQIYARYFLERLSNDLPDVYPFYYSKPVKSAYNPNLRYHNGEEMPVRPSNLYVTNFDLYYIADIKNYEKRVEDAIDFGYVFDEHVKPHSLYHDVHGMEYVADMIEGNMDSPNFYFYGSIYHMYHSMIGHIVDPYHKMGLAPSALEHPETVLRDPAFYQLWKRVDHLFQKYKNRLPRYTHDELAFEGVKVENVDVGKLYTYFEQYDVSLDMSVYVNKVDQIPNVDVHARQYRLNHKPFTYNIEVSSDKAQDVYVRVFLGPKYDYLGREYDLNDRRHYFVEMDRFPHHVEAGKTVIERNSHDSNIVAPERDSYRTFYKKVQEAYEGKSQYYVDKGHNYCGYPENLLIPKGKKGGQAYTFYVIVTPYVKQDEHDFEPYNYKAFSYCGVGSNRKYPDNMPLGYPFDRKIYSNDFYTPNMYFKDVIIFHKKYDEVGVQGH

| **Position** | **Residue** | **Score^*^** | **Jury agreement^∞^** | **N-Glyc result^$^** |
| --- | --- | --- | --- | --- |
| **199** | **N**FT | 0.6672 | (8/9) | + |
| **234** | **N**TT | 0.6939 | (9/9) | ++ |

**^*^**The score is the averaged output of nine neural networks.

**^∞^**The jury agreement column indicates how many of the nine networks support the prediction.

**^$^**+ Score > 0.5; ++ Score > 0.5 and Jury agreement (9/9) or Score >0.75; +++ Score > 0.75 and Jury agreement (9/9) ; ++++ Score > 0.90 and Jury agreement (9/9).

**Supplementary Table S9: Potential N-linked glycosylation sites for HBV PLA2/Api m 1.**

>Phospholipase A-2 Length = 162

GSLFLLLLSTSHGWQIRDRIGDNELEERIIYPGTLWCGHG**N**KSSGPNELGRFKHTDACCRTHDMCPDVMSAGESKHGLTNTASHTRLSCDCDDKFYDCLKNSADTISSYFVGKMYFNLIDTKCYKLEHPVTGCGERTEGRCLHYTVDKSKPKVYQWFDLRKY

| **Position** | **Residue** | **Score^*^** | **Jury agreement^∞^** | **N-Glyc result^$^** |
| --- | --- | --- | --- | --- |
| **41** | **N**KS | 0.6746 | (8/9) | + |

**^*^**The score is the averaged output of nine neural networks.

**^∞^**The jury agreement column indicates how many of the nine networks support the prediction.

**^$^**+ Score > 0.5; ++ Score > 0.5 and Jury agreement (9/9) or Score >0.75; +++ Score > 0.75 and Jury agreement (9/9) ; ++++ Score > 0.90 and Jury agreement (9/9).

**Supplementary Table S10: Potential O-linked glycosylated sites for PLA2/Api m 1.**

>Phospholipase A-2 Length = 162

GSLFLLLLSTSHGWQIRDRIGDNELEERIIYPGTLWCGHGNK**S**SGPNELGRFKH**T**DACCRTHDMCPDVMSAGESKHGLTN**T**ASHTRLSCDCDDKFYDCLKNSADTISSYFVGKMYFNLIDTKCYKLEHPVTGCGERTEGRCLHYTVDKSKPKVYQWFDLRKY

| **Position** | **Residue** | **Score^*^** | **Prediction** |
| --- | --- | --- | --- |
| **43** | **S** | 0.5 | O-Glycosylated |
| **55** | **T** | 0.522442 | O-Glycosylated |
| **81** | **T** | 0.550287 | O-Glycosylated |

**^*^** Residues with scores ≥ 0.5 were predicted as glycosylated.
